# Circular RNA circDtx1 regulates IRF3-mediated antiviral immune responses through suppression of miR-15a-5p-dependent TRIF downregulation in teleost fish

**DOI:** 10.1371/journal.ppat.1009438

**Published:** 2021-03-18

**Authors:** Weiwei Zheng, Qing Chu, Liyuan Yang, Lingping Sun, Tianjun Xu

**Affiliations:** 1 Laboratory of Fish Molecular Immunology, College of Fisheries and Life Science, Shanghai Ocean University, Shanghai, China; 2 Laboratory of Marine Biology and Biotechnology, Qingdao National Laboratory for Marine Science and Technology, Qingdao, China; 3 Key Laboratory of Exploration and Utilization of Aquatic Genetic Resources (Shanghai Ocean University), Ministry of Education, Shanghai, China; 4 National Pathogen Collection Center for Aquatic Animals, Shanghai Ocean University, Shanghai, China; Stanford University, UNITED STATES

## Abstract

Circular RNAs (circRNAs) represent a class of widespread and diverse covalently closed circular endogenous RNAs that exert crucial functions in regulating gene expression in mammals. However, the function and regulation mechanism of circRNAs in lower vertebrates are still unknown. Here, we discovered a novel circRNA derived from Deltex E3 ubiquitin ligase 1 (*Dtx1*) gene, namely, circDtx1, which was related to the antiviral responses in teleost fish. Results indicated that circDtx1 played essential roles in host antiviral immunity and inhibition of SCRV replication. Our study also found a microRNA miR-15a-5p, which could inhibit antiviral immune response and promote viral replication by targeting TRIF. Moreover, we also found that the antiviral effect inhibited by miR-15a-5p could be reversed with the circDtx1. In mechanism, our data revealed that circDtx1 was a competing endogenous RNA (ceRNA) of TRIF by sponging miR-15a-5p, leading to activation of the NF-κB/IRF3 pathway, and then enhancing the innate antiviral responses. Our results indicated that circRNAs played a regulatory role in immune responses in teleost fish.

## Author summary

Increasing evidence indicates that circRNAs participate in innate and adaptive immunity. However, the relationship between circRNAs and host antiviral responses remains unknown, particularly in lower vertebrates. Our results provided direct evidence that a circRNA, namely, circDtx1, is related to the antiviral responses in lower vertebrates. In addition, our study also found a microRNA, namely, miR-15a-5p, which could inhibit an antiviral immune response and promote viral replication by targeting TRIF. Moreover, circRNAs can serve as competing endogenous RNAs (ceRNAs) and cross-talk with mRNAs by competing shared miRNAs. Such ceRNAs regulate the distribution of miRNA molecules on their targets and apply an additional level of post-transcriptional regulation. In our study, circDtx1 functions as a ceRNA for miR-15a-5p to control protein abundance of fish TRIF, thereby inhibiting viral replication and promoting antiviral responses. Our study demonstrates the ceRNA regulatory networks existing in lower vertebrates, which can provide new insights into understanding the effects of circRNAs on host-virus interactions.

## Introduction

Innate immunity, as the host’s first line of defense against external pathogens, rapidly responds to invading viruses, which primarily rely on its possession of multiple pattern recognition receptors (PRRs) that rapidly recognize various viral pathogen associated molecular patterns (PAMPs) and transmit signals to activate downstream antiviral immune signaling pathways. This process enables the host to produce type I interferon (IFN) and IFN-stimulating genes (ISG) in time to clear the infection of the virus [1]. Toll-like receptors (TLRs) and retinoic acid-inducible gene-I (RIG-I)-like receptors (RLRs) are two kinds of PRRs, which primarily act as virus sensors [1, 2]. Unlike RLRs, which are primarily responsible for the surveillance of intracellular viruses, TLRs are primarily responsible for the recognition of extracellular viruses or viruses that enter the endosome by endocytosis [3, 4]. In addition, different TLRs family members can work together to identify viruses and activate antiviral immune signaling pathways by monitoring different components of the virus, including viral protein components, RNA (ssRNA and dsRNA), and DNA [5–8]. After PRRs recognize the virus, host cells need specific adaptor proteins to transmit signals and activate downstream signaling pathways [9]. Toll–interleukin 1 receptor domain-containing adaptor molecule (TICAM-1, also called TRIF) is an important adaptor protein, which plays an irreplaceable role in the process of host antiviral immunity [10–13].

TLR3, one of the most important members of the TLR family, has an important role in antiviral immune responses, whereas TLR3 has also been found to be the only one among all the identified TLRs that does not rely on myeloid differentiation factor 88 (MyD88) to mediate immune responses. As a dsRNA virus monitor, TLR3-mediated antiviral immune response depends on TRIF. After TLR3 recognizes the virus, it can quickly recruit specific downstream molecular adapter TRIF through the TIR domain, and migrate in a vesicle-like structure to form the TRIF signal body structure, which promotes the activation of downstream antiviral immune response and IFN production [14–16]. Unlike mammals, fish has a fish-specific TLR22, in addition to TLR3 being involved in the recognition of viruses and eliciting immune responses, which can participate in the recognition of RNA viruses, and it is necessary for the recruitment of the adaptor molecule TRIF when mediating antiviral immune responses [14]. After finding that TRIF is a bridge-mediating antiviral immune response, researchers have realized that the regulation of TRIF will have a great impact on antiviral immunity; thus researchers paid great attention to the regulation of TRIF. In the past few decades, many proteins have been found to regulate the TRIF-mediated signaling pathway in human. For example, selective androgen receptor modulators adaptor can negatively regulate TLR signal mediated by an adaptor protein, namely, TRIF [17]. Furthermore, the disintegrin and metalloprotease 15 can regulate TRIF-dependent TLR3 and TLR4 signal transduction by mediating the degradation of TRIF, thereby protecting the host from excessive production of pro-inflammatory cytokines and matrix metalloproteinases [18]. In addition to the regulators that can regulate TRIF in mammals, Zhao et al. has recently found a class of regulatory factors that can negatively regulate the TRIF-mediated NF-kB signaling pathway in teleost. They have found that interferon regulatory factor 3 (IRF3) and IRF9 can serve as negative regulators of TRIF to regulate its mediated immune response and prevent the excessive immune response of the body [19, 20]. Despite considerable research on mammalian TRIF in antiviral immunity and anti-tumor, research on TRIF in lower vertebrates still has many challenges; therefore, exploration of the regulatory mechanism of TRIF-mediated signal transduction is urgently needed.

Non-coding RNA (ncRNA) is defined as a collection of RNAs that do not have the potential to encode proteins [21]. microRNAs (miRNAs), long non-coding RNAs (lncRNAs), and circular RNAs (circRNAs) are the three types of non-coding RNAs that have been studied comprehensively in mammals [22–24]. miRNAs are a class of highly conserved ncRNAs with a length of only 21–24 nucleotides [25], which can participate in various physiological and pathological development processes (such as regulating cell proliferation, growth, apoptosis, and differentiation) and has been widely recognized and confirmed [26]. Through binding to the 3’-untranslated region (UTR) of the target mRNAs, miRNAs repress gene expression by inhibiting mRNA translation or promoting mRNA degradation. Recent studies have shown that miRNAs play pivotal roles in regulating virus-induced immune response among different vertebrate species. For example, in mammals, miR-146a and miR-144 have been reported to involve in regulating antiviral responses upon vesicular stomatitis virus infection [27, 28]. In addition, miR-23a and miR-101 reduce the host’s antiviral activity by targeting interferon regulatory factor 1 and mitochondrial ATP synthase subunit beta upon herpes simplex virus-1 infection [29, 30]. In birds, miR-9* has been shown to target IRF1 and downregulate the antiviral responses in infectious bursal disease viral infection [31]. Most recently, in lower vertebrates, studies have reported that fish miRNAs, such as miR-122, miR-210, and miR-3570, have a negative regulatory effect on the regulation of antiviral innate immune responses upon rhabdovirus infection [32–34].

circRNA, a non-coding RNA, is a covalently closed circular molecule generated by back-splicing, and it is first found in the hepatitis D virus in the 1970s [35]. At first, circRNA was thought to be a mistake in the splicing of exons or introns or intermediate escaping from the decoupled branches of introns [36]. With the development of high-throughput sequencing technology and bioinformatics, an increasing number of circRNAs have been identified in eukaryotes [37, 38]. At present, back-splicing is specific splicing widely found in various eukaryotes. According to the existing theories, the generation of circRNA is different from the canonical eukaryotic pre-mRNA splicing, and back-splicing reverses the downstream splice donor site and upstream splice acceptor site, resulting in a covalently closed circular RNA transcript and alternative splicing of linear RNA, thereby skipping the exon [37, 39]. This finding indicates that circRNA does not have the 5 ’ cap and 3’ polyadenylation as traditional linear mRNA; therefore, circRNA has higher stability than linear RNA. Given the characteristic of circRNA, RNase R with 3’ to 5’ exonuclease activity has become one of the most convenient ways to enrich circRNA [40]. To date, circRNA has been reported to participate in various biological processes, including proliferation, invasion, and metastasis [41, 42]. Considerable evidence has shown that circRNA can serve as a miRNA sponge to competitively adsorb miRNA to regulate its target genes, thereby forming a competing endogenous RNAs (ceRNAs) regulatory network of circRNA-miRNA-mRNA, which is similar to lncRNA [38]. For example, as the first circRNA reported as ceRNA, ciRC-7 contains many conserved sequences of miR-7, which directly targets several oncogenes and involves different human cancers. Therefore, ciRC-7 can indirectly regulate cancer by inhibiting the activity of miR-7 [43]. Since then, increasing reports about circRNA acting as a miRNA molecular sponge in mammals have been found, but whether the mechanism exists in lower vertebrates, such as fish, remains unknown.

In this study, we identify a ceRNA regulatory network involved in antiviral responses in teleost fish, namely, miiuy croaker (*Miichthys miiuy*). We elaborate that fish TRIF contributes to IFN antiviral immunity following the infection of *Siniperca chuatsi rhabdovirus* (SCRV), a typical fish RNA rhabdovirus. Here, we have found that miR-15a-5p target TRIF and suppress TRIF-mediated antiviral responses, thereby promoting RNA viral replication. Furthermore, our study suggests that a circular RNA, namely, circular RNA Dtx1 (circDtx1), can serve as a ceRNA for miR-15a-5p to facilitate TRIF expression, thereby modulating TRIF-mediated antiviral responses and suppressing viral replication. Our results not only elucidate the biological mechanism of the circRNA-miRNA-mRNA axis in antiviral immune responses of fish but also provide a new idea for the study of immune regulation in lower vertebrates.

## Results

### Characterization of CircDtx1 involved in antiviral immunity

**Fig 1:**
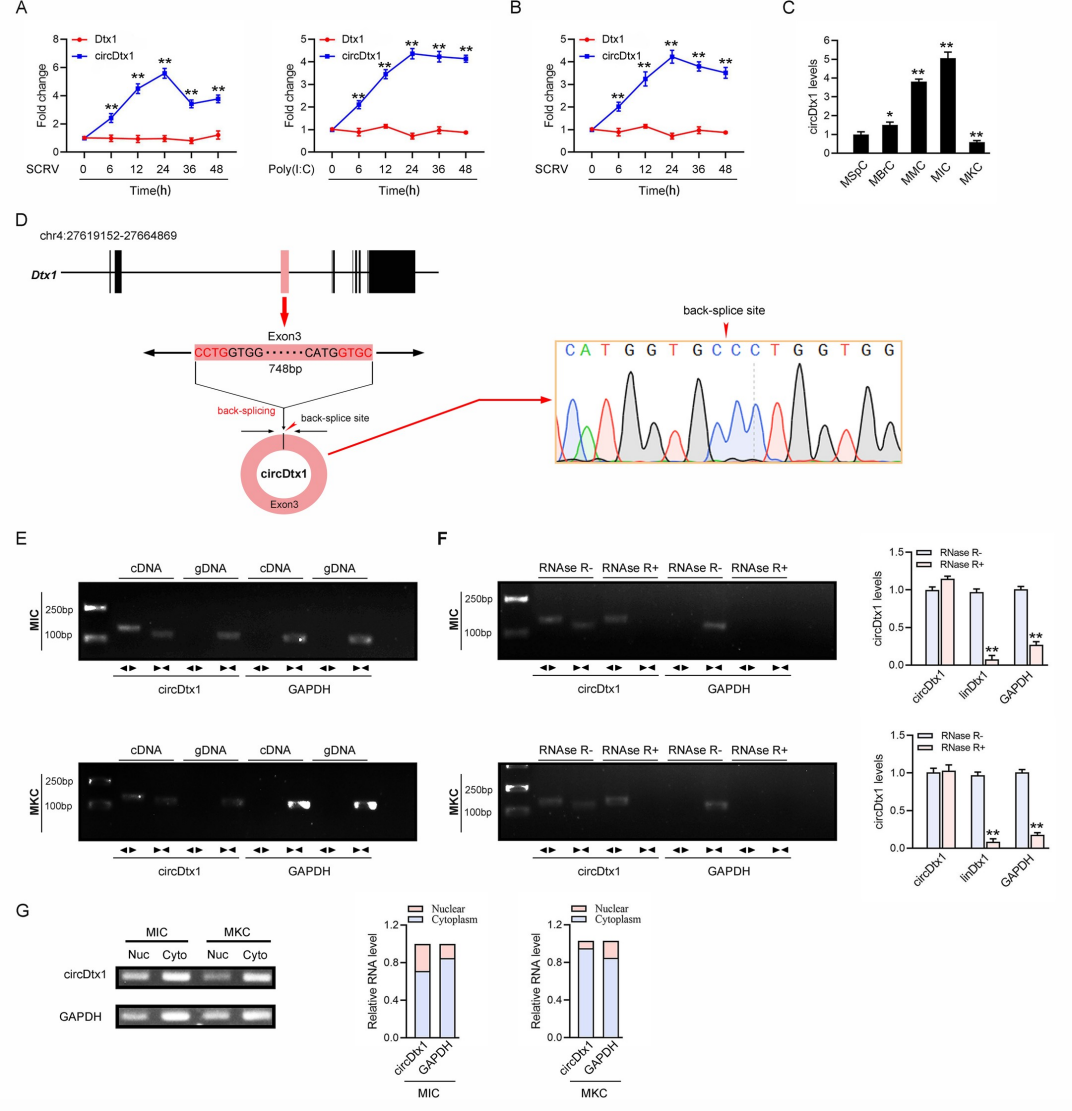
Expression profiles and characterization of circDtx1. (A) qPCR for the abundance of circDtx1 and linear *Dtx1* (Dtx1) mRNA in spleen tissues treated with SCRV (MOI = 5) and poly(I:C) at the indicated time points, respectively. (B) qPCR analysis of circDtx1 and linear *Dtx1* mRNA in MKC cells treated with SCRV (MOI = 5) at the indicated time points. (C) Relative expression of circDtx1 in indicated cell lines was determined by qPCR. (D) We confirmed the head-to-tail splicing of circDtx1 in the circDtx1 RT-PCR product by Sanger sequencing. (E) RT-PCR validated the existence of circDtx1 in MIC and MKC cell lines. circDtx1 was amplified by divergent primers in cDNA but not gDNA. GAPDH was used as a negative control. (F) The expression of circDtx1 and linear *Dtx1* mRNA in both MIC and MKC cell lines was detected by RT-PCR assay followed by nucleic acid electrophoresis or qPCR assay in the presence or absence of RNase R. (G) circDtx1 was mainly localized in the cytoplasm. RNA isolated from nuclear and cytoplasm was used to analyze the expression of circDtx1 by RT-PCR; n = 3. The data represented the mean ± SD from three independent triplicated experiments. **, *p* < 0.01.

A large number of circRNAs were involved in the organism’s antiviral immune responses in mammals [10], but the role of circRNAs in the immune responses in lower vertebrates remained unclear. We used RNA-seq data to compare the expression levels of circRNA after SCRV infection and then it was found that the expression of circDtx1 was significantly up-regulated after SCRV infection. We treated miiuy croaker with SCRV and poly (I:C) to further confirm the reliability of RNA-seq data, sampled tissues at different times to extract RNA, and then quantitatively analyzed the expression level of circDtx1 by quantitative real-time polymerase chain reaction (qPCR). In addition, considering that circRNAs were produced by linear RNA splicing, the expression levels of linear Deltex E3 ubiquitin ligase 1 (*Dtx1*) and circDtx1 were also detected. The qPCR results confirmed that circDtx1 was significantly upregulated under SCRV and poly (I:C) stimulation compared with linear *Dtx1* (Fig 1A). In addition, SCRV-treated miiuy croaker kidney cells (MKC) further confirmed the significant expression of circDtx1 (Fig 1B). We then evaluated the expression levels of circDtx1 in miiuy croaker spleen cells, miiuy croaker brain cells, miiuy croaker muscle cells, miiuy croaker intestine cells (MIC), and MKC (Fig 1C). Among the aforementioned cell lines, MIC and MKC cells showed the highest and the lowest expression of circDtx1, respectively. Therefore, we selected MIC and MKC to investigate the function and regulatory mechanism of circDtx1.

We blasted the *Dtx1* gene with the whole genome of the miiuy croaker and found that the *Dtx1* gene was located on chromosome 4. circDtx1 was consisted of the head-to-tail splicing of only exon 3, with a spliced mature sequence length of 748 bp (S1 Fig). We used several universal circRNAs detection methods to distinguish whether the head-to-tail splicing is the result of trans-splicing or the genome rearrangement. We first designed divergent primers to amplify circDtx1, and the result of Sanger sequencing confirmed the head-to-tail splicing in the RT-PCR product of circDtx1 (Fig 1D). Then, we used convergent primers to amplify *Dtx1* gene and divergent primers to amplify circDtx1. cDNA and gDNA were extracted separately from MKC and MIC and subjected to RT-PCR and agarose gel electrophoresis assays. The results shown in Fig 1E indicated that circDtx1 was amplified from cDNA by using only divergent primers (an expected 145 bp fragment), whereas no amplification product was observed from gDNA. Considering that stability was a crucial characteristics of circRNAs, we thus employed RNase R to confirm the stability of circDtx1. The results from the analysis of RT-PCR and agarose gel electrophoresis assay showed that circDtx1, rather than linear *Dtx1* or GAPDH, resisted digestion by RNase R (Fig 1F). In addition, we detected the distribution of circDtx1 by cytoplasmic nuclear fractionation experiments and found that circDtx1 was primarily localized in the cytoplasm (Fig 1G). Accordingly, these results suggested that circDtx1 was a stable circRNA expressed and primarily distributed in the cytoplasm.

### circDtx1 enhances host antiviral innate immunity

**Fig 2:**
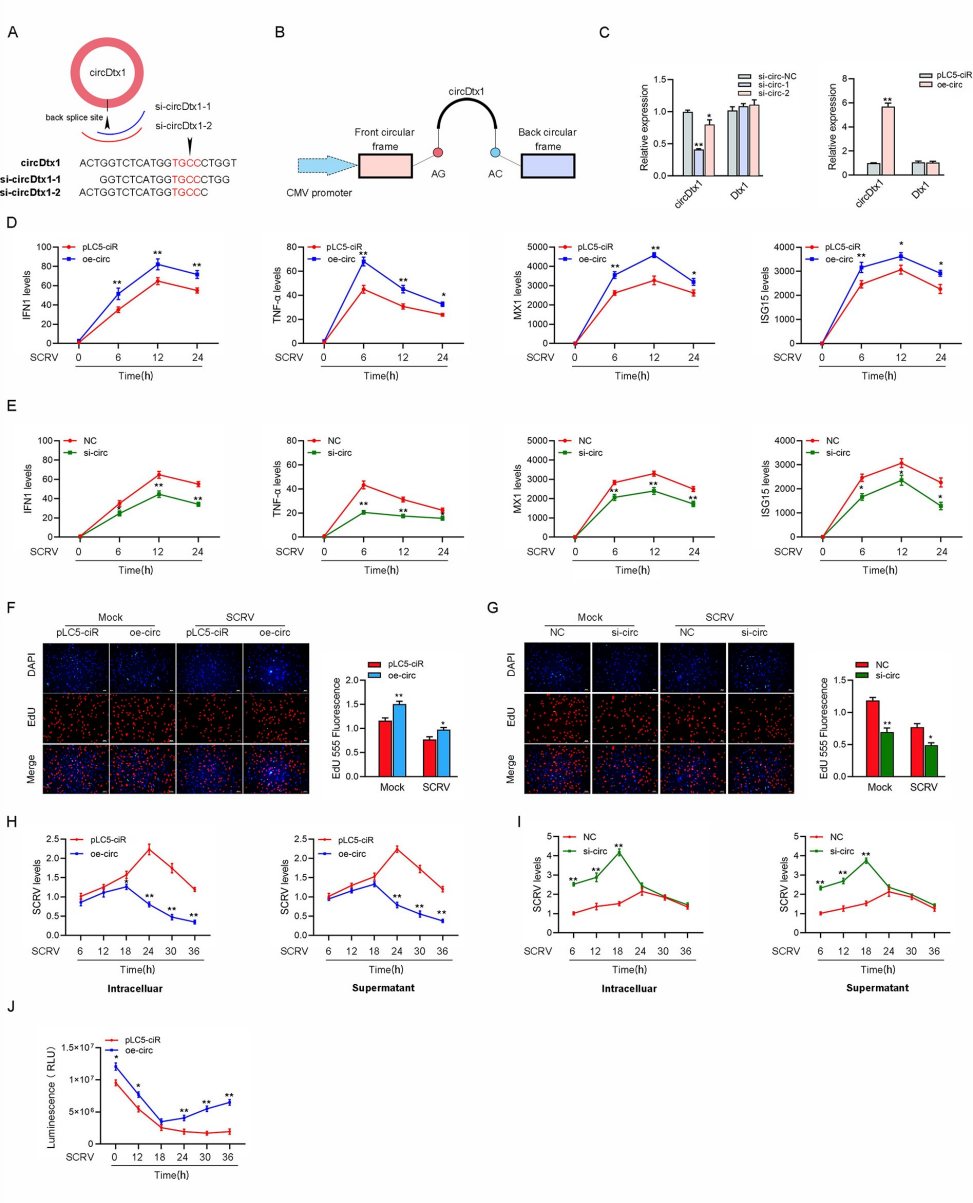
circDtx1 promotes the antiviral innate immunity. (A and B) The schematic diagram of siRNAs (A) and oe-circ structure (B) qPCR analysis of circDtx1 and linear Dtx1 mRNA in MIC cells treated with siRNAs. (C) qPCR analysis of circDtx1 and linear Dtx1 mRNA in MKC cells stably overexpressing circDtx1. (D and E) qPCR assays were performed to determine the expression levels of IFN1, TNF-α, MX1, and ISG15 in MKC cells transfected with overexpression plasmid (oe-circ) or control vector (pLC5-ciR) (D) and MIC cells transfected with (si-circDtx1-1) si-circ or NC (E). (F and G) Cell proliferation was assessed by EdU assays in MKC cells transfected with oe-circ or pLC5-ciR vector after SCRV infected 24 h (F) and MIC cells transfected with si-circ or NC after SCRV infected 18 h (G). (H and I) circDtx1 suppresses SCRV replication. MKC cells transfected with pLC5-ciR vector or oe-circ plasmid (H) and MIC were transfected with NC or si-circ (I) for 24 h, respectively, then infected with SCRV at different time. The qPCR analysis was conducted for intracellular and supernatant SCRV RNA expression. (J) Effect of circDtx1 on cell viability after SCRV infection. MIC cells were transfected with pLC5-ciR vector or oe-circ for 24 h, then treated with SCRV for different times. Cell viability assays were measured. All data represented the mean ± SD from three independent triplicated experiments. *, *p* < 0.05; **, *p* < 0.01.

The small interfering RNAs (siRNA) against circDtx1 and the overexpression plasmid of circDtx1 were constructed to detect the biological function of circDtx1 (Fig 2A and 2B). Consequently, two siRNAs (si-circDtx1-1 and si-circDtx1-2) evidently decreased the circDtx1 expression level, but such siRNAs did not affect the expression level of linear *Dtx1* mRNA in MIC. As si-circDtx1-1 could induce higher inhibitory efficiency; thus we selected si-circDtx1-1(si-circ-1) for the subsequent experiment (left panel of Fig 2C). Moreover, the circDtx1 overexpression plasmid was successfully constructed, as it significantly increased the circDtx1 expression levels rather than linear *Dtx1* mRNA in MKC (right panel of Fig 2C). Considering that IFN and ISGs are important antiviral effectors, we focused on investigating the role of circDtx1 in regulating the expression of IFN, ISGs, and inflammatory cytokines. As shown in Fig 2D, the overexpression of circDtx1 could significantly inhibit the expression levels of interferon IFN1, inflammatory cytokines (TNF-α), and antiviral genes such as myxovirus resistance protein 1 (MX1) and ISG15 after SCRV infection. By contrast, knockdown of circDtx1 (oe-circ) increased the expression levels of these genes under SCRV treatment (Fig 2E). We conducted EdU assays to examine the cell proliferation in MIC and MKC and explore the function of circDtx1 in antiviral innate immunity. The results showed that knockdown of circDtx1 considerably decreased the percentages of EdU-positive cells (Fig 2F) but greatly increased at overexpression of circDtx1, suggesting that circDtx1 promoted the proliferation of miiuy croaker cell lines (Fig 2G). Furthermore, we examined the effect of circDtx1 on SCRV replication to explore the biological significance of circDtx1 in SCRV-induced host cells. Detecting SCRV RNA level by qPCR, we found that circDtx1 overexpression significantly inhibited SCRV replication after 18h of SCRV infection (Fig 2H), while circDtx1 knockdown significantly promoted SCRV replication before 24h of SCRV infection (Fig 2I). When we investigated the effect of circDtx1 on the cell viability of MKC, we found that overexpression of circDtx1 significantly increased cell viability compared with the control group after 24 h of SCRV infection (Fig 2J). In summary, these data indicate that circDtx1, as a positive regulator, is involved in the regulation of antiviral immunity, and the data of the cell proliferation and viability suggestion that the circDtx1 can positively regulate the antiviral responses and upregulate the expression of inflammatory cytokines and antiviral genes, reducing the attack of the virus to cells, and promoting cell proliferation and increasing cell viability.

### circDtx1 is able to regulate miR-15a-5p expression and activity

**Fig 3:**
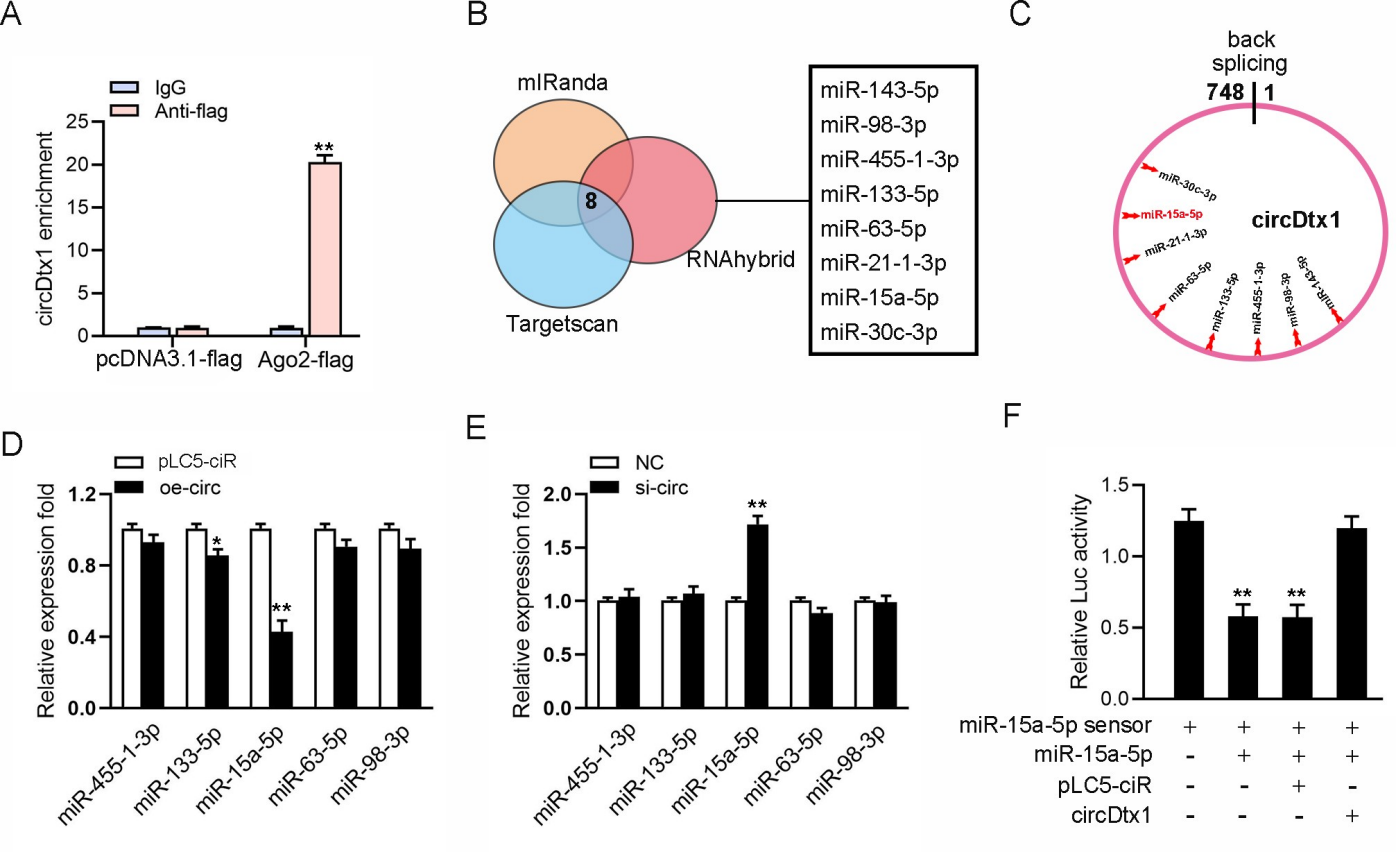
circDtx1 regulates miR-15a-5p expression and activity. (A) The Ago2-RIP assay for the amount of circDtx1 in MIC cells transfected Ago2-flag or pcDNA3.1-flag. (B) A schematic illustration showing overlapping of the target miRNAs of circDtx1 predicted by TargetScan, mIRanda, and RNAhybrid. (C) Schematic drawing showing the putative binding sites of the miRNAs associated with circDtx1. (D and E) Relative expression of candidate miRNAs in MIC and MKC cells transfected with oe-circ (D) and si-circ (E), respectively. (F) circDtx1 reduces miR-15a-5p activity. The relative luciferase activity was analyzed in MKC cells co-transfected with mimics, circDtx1 overexpression plasmid, and control vector, together with miR-15a-5p sensor. All data represented the mean ± SD from three independent triplicated experiments. **, *p* < 0.01.

We examined the ability of circDtx1 to bind to miRNAs to explore whether circDtx1 can function as a miRNA sponge. To this end, we transfected Ago2-flag or pcDNA3.1-flag into MIC cells to conduct RNA immunoprecipitation (RIP) for Argonaute (Ago2). The results showed that endogenous circDtx1 could be pulled down by Ago2-flag (Fig 3A), indicating that circDtx1 might have a binding site with miRNA. Next, in finding miRNAs combined with circDtx1, we first used miRNA target prediction tools including TargetScan, miRanda, and RNAhybrid for prediction, and selected five candidate miRNAs for further verification (Fig 3B and 3C). Afterward, we compared the expression levels of these candidate miRNAs in MIC transfected with si-circ or negative control and MKC cells transfected with overexpression plasmid (oe-circ) or control vector (vector). Among the five candidate miRNAs, miR-15a-5p expression was significantly reduced when circDtx1 was overexpressed (Fig 3D), whereas miR-15a-5p expression was significantly enhanced in response to circDtx1 inhibition compared with other candidate miRNAs (Fig 3E). We constructed the miR-15a-5p sensor to detect whether circDtx1 affects the activity of miR-15a-5p and consolidate the direct binding of miR-15a-5p and circDtx1. Then, we transfected the miR-15a-5p sensor with miR-15a-5p, pLC5-ciR vector, or circDtx1 overexpression plasmid. The decreased luciferase activity induced by miR-15a-5p was recovered when cotransfected with circDtx1 overexpression plasmid, suggesting that circDtx1 specifically sponged miR-15a-5p, thereby preventing it from inhibiting luciferase activity (Fig 3F). Collectively, circDtx1 could regulate miR-15a-5p expression and activity, and circDtx1 might function as a sponge of miR-15a-5p.

### cicrDtx1 functions as a miRNA sponge of miR-15a-5p

**Fig 4:**
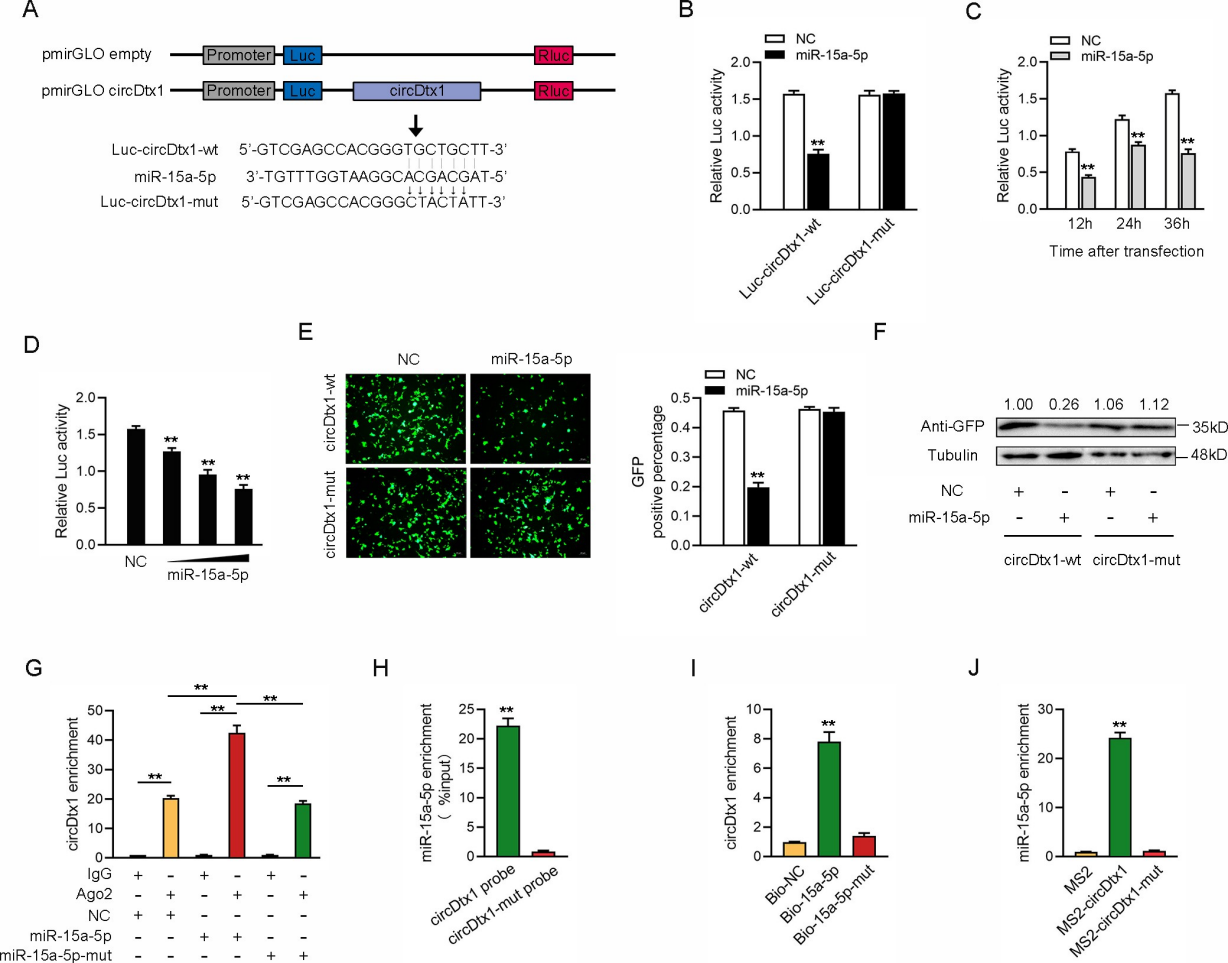
circDtx1 functions as a miRNA sponge of miR-15a-5p. (A) Schematic illustration of circDtx1-wt and circDtx1-mut luciferase reporter vectors. (B) The relative luciferase activities were detected in EPC cells after cotransfection with circDtx1-wt or circDtx1-mut and mimics or NC. (C and D) The concentration gradient (C) and time gradient (D) experiment of miR-15a-5p mimics was conducted. (E and F) circDtx1 downregulated GFP expression. EPC cells were cotransfected with circDtx1-wt or circDtx1-mut and mimics or NC. The fluorescence intensity and the GFP expression were evaluated by enzyme-labeled instrument and western blotting, respectively. (G) The Ago2-RIP assay was executed in MIC cells after transfection with miR-15a-5p, miR-15a-5p-mut, and NC, followed by qPCR to detect cirDtx1 expression levels. (H and I) RNA pull-down assay was executed in MIC cells, followed by qPCR to detect the enrichment of circDtx1 and miR-15a-5p. (J) The MS2-RIP assay was executed in MIC cells after transfection with pLC5-circ-MS2, pLC5-MS2-circDtx1, pLC5-MS2-circDtx1-mut, followed by qPCR to detect the enrichment of miR-15a-5p. All data represented the mean ± SD from three independent triplicated experiments. *, *p* < 0.05; **, *p* < 0.01.

We analyzed the sequences of circDtx1 to investigate whether circDtx1 could interact with miR-15a-5p and found that circDtx1 contained a binding site of miR-15a-5p (Fig 4A). Next, we constructed a luciferase plasmid of circDtx1 and the mutated form of miR-15a-5p binding sites mutated (Fig 4A). Luciferase assays revealed that miR-15a-5p could suppress the luciferase activity of the wild form of circDtx1 luciferase plasmid, but it had no effect on the mutated form (Fig 4B). In addition, miR-15a-5p mimics inhibited luciferase activity in time-dependent and dose-dependent manner (Fig 4C and 4D). Moreover, we inserted a wild or a mutated form of circDtx1 into the mVenus-C1 vector and examined whether cotransfecting with miR-15a-5p could suppress the levels of green fluorescent protein (GFP). As shown in Fig 4E and 4F, the results revealed that miR-15a-5p could significantly inhibit the levels of GFP, which suggested that a direct interaction might exist between circDtx1 and miR-15a-5p.

Given that miRNAs regulated target gene expression by binding to Ago2, we further tested the ability of circDtx1 to bind to miR-15a-5p. To this end, RIP assays were performed in MIC cells by cotransfecting Ago2-flag, miR-15a-5p, and miR-15a-5p-mut. The results from qPCR analysis indicated that circDtx1 and miR-15a-5p were efficiently pulled down by Ago2-flag, but not miR-15a-5p-mut (Fig 4G). We conducted RNA pulldown assays with biotin-labeled circDtx1 probe or biotin-labeled miR-15a-5p to further confirm the direct interaction between circDtx1 and miR-15a-5p. The results from the qPCR analysis revealed that miR-15a-5p could be pulled down by biotin-labeled circDtx1 but not circDtx1-mut (Fig 4H). Additionally, biotin-labeled miR-15a-5p captured more circDtx1 compared with the negative control and biotin-labeled miR-15a-5p-mut (Fig 4I). Furthermore, RIP assays were applied to test the direct interaction between circDtx1 and miR-15a-5p. We cloned an MS2 fragment into pLC5-ciR, pLC5-circDtx1, and pLC5-circDtx1-mut plasmids to construct plasmids that could produce circDtx1 identified by the MS2 protein. We also constructed a GFP and MS2 gene fusion expression vector to produce a GFP-MS2 fusion protein that could bind with the MS2 fragment and be identified using an anti-GFP antibody. Hence, miRNAs that interacted with circDtx1 could be pulled down by the GFP-MS2-circDtx1 compounds. The results from qPCR assays showed that the pLC5-circDtx1 RIP was significantly enriched for miR-15a-5p compared with pLC5-circDtx1-mut or empty vector (Fig 4J). Collectively, these data demonstrated that circDtx1 could directly bind to miR-15a-5p, and circDtx1 served as a sponge of miR-15a-5p.

### Fish TRIF enhance antiviral responses upon SCRV infection

**Fig 5:**
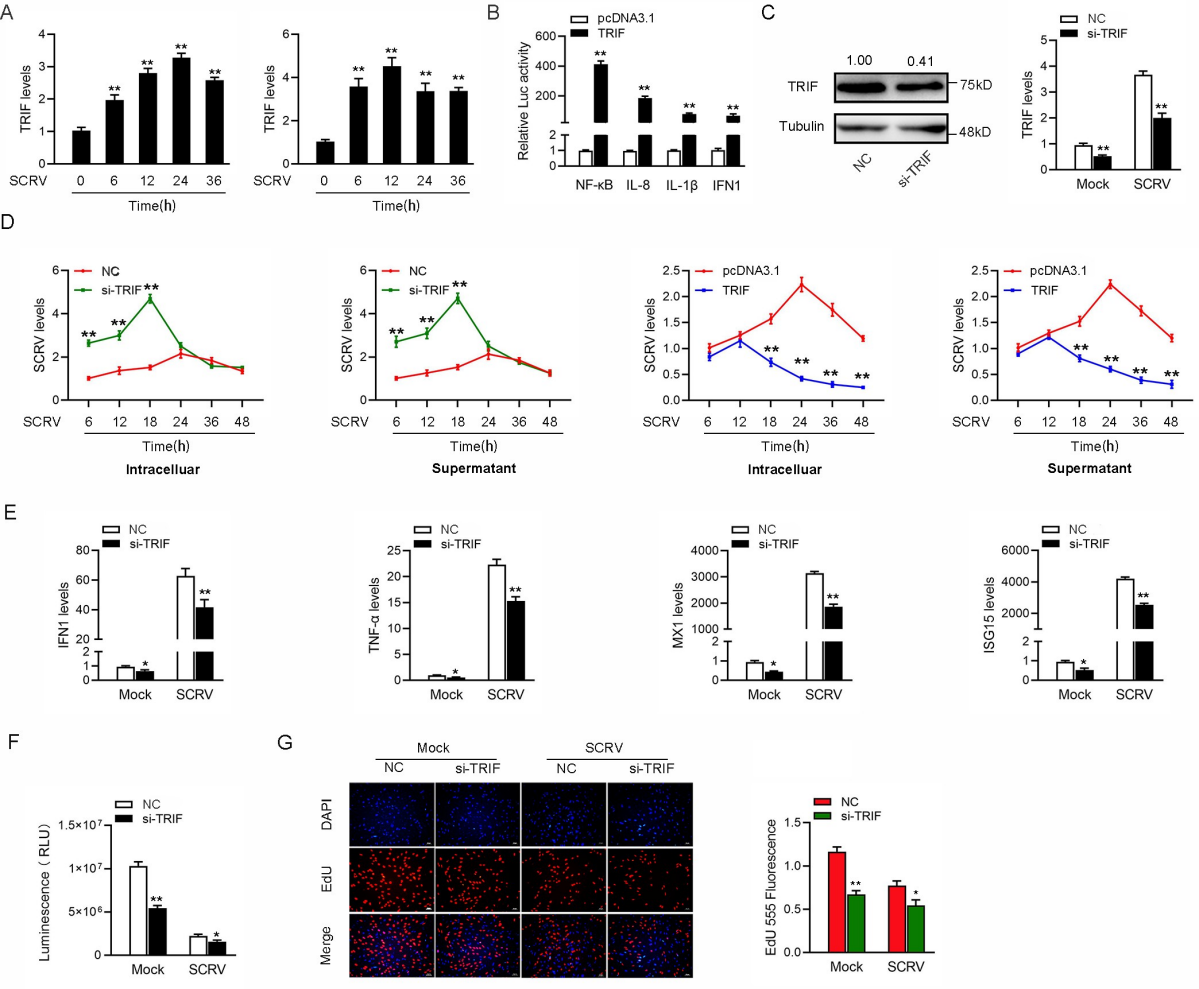
Fish TRIF suppresses antiviral responses upon SCRV infection. (A) SCRV induces an increase in TRIF expression. The expression levels of TRIF in MIC cells and intestine samples were measured by qPCR at indicated time after SCRV infection. (B) TRIF is able to activate NF-κB, IL-8, IL-1β, and IFN1 signaling. MIC cells were transfected with phRL-TK Renilla luciferase plasmid, luciferase reporter genes, together with TRIF expression plasmid. At 48 h post-transaction, the luciferase activity was measured and normalized to renilla luciferase activity. (C) Knockdown of TRIF attenuates the expression of endogenous TRIF. MIC cells were transfected with si-Ctrl or si-TRIF for 48 h, then the expression levels of TRIF were determined by western blotting and qPCR assays, respectively. (D) Fish TRIF suppresses SCRV replication. MIC cells were transfected with pcDNA3.1 vector or TRIF expression plasmid and control siRNA (si-Ctrl) or TRIF-specific siRNA (si-TRIF) for 24 h, then infected with SCRV for different times. The qPCR analysis was conducted for intracellular and supernatant SCRV RNA expression. (E) Knockdown of TRIF attenuates the expression of antiviral genes. MIC cells were transfected with si-Ctrl or si-TRIF. At 24 h post-transfection, cells were then treated with SCRV for 24 h. The expression of IFN1, TNF-α, Mx1, and ISG15 were determined by qPCR. (F and G) Effect of TRIF knockdown on cell proliferation and viability after SCRV infection. MIC cells were transfected with either si-TRIF or si-Ctrl. At 24 h post-transfection, the cells were infected with SCRV for 24 h, then cell viability assay (F) and cell proliferation assay (G) were measured. Scale bar, 20 μm. All data represented the mean ± SD from three independent triplicated experiments. *, *p* < 0.05; **, *p* < 0.01.

Viral infection triggers host innate immune responses by activating transcription factors, namely, IRF3 and NF-κB, which coordinately induce the production of type I IFNs. Mammal TRIF is known as an essential signaling adaptor involved in host antiviral innate immunity in response to dsRNA viral infection. Recent findings suggest that TRIF homolog genes have been identified in some fish species. However, the signaling pathway involved in fish TRIF-mediated immune response remains poorly understood. In investigating the fish TRIF-mediated signaling pathway in response to RNA viral infection, we firstly examined the expression patterns of fish TRIF upon SCRV. To this end, we treated MIC with SCRV or the intestinal tissue of miiuy croaker infected by SCRV. During SCRV infection, the expression levels of TRIF were significantly increased in vitro and in vivo (Fig 5A). Given that mammal TRIF activated NF-κB and IRF3 to induce IFNs, we tested whether fish TRIF could affect the activation of NF-κB and IRF3. The results from dual-luciferase reporter assays showed that overexpression of TRIF potently activates NF-κB and IFN1 reporter genes, as well as IL-8 and IL-1β reporter genes (Fig 5B). We silenced TRIF and examined the expression patterns of indicated genes to confirm whether fish TRIF is required for the induction of type I IFN and inflammatory cytokines upon SCRV infection. Knockdown of TRIF effectively inhibited TRIF expression at protein and mRNA levels (Fig 5C).

Considering that mammal TRIF overexpression was sufficient to delay viral replication, we determined whether fish TRIF could mediate a similar effect upon RNA viral infection. The results indicated that overexpression of TRIF decreased SCRV replication in the infected cells, whereas TRIF-specific siRNA exacerbated the viral replication to silence the expression of endogenous TRIF (Fig 5D). These results suggested that similar to mammal TRIF, TRIF in teleost fish could mediate the activation of NF-κB and IRF3 and modulate RNA viral replication. Furthermore, as shown in Fig 5E, knockdown of TRIF significantly decreased the expression of IFN1, antiviral genes, and inflammatory cytokines, including TNF-α, Mx1, and ISG15 in MIC under SCRV treatment. The result indicated the contribution of TRIF to fish antiviral responses in response to RNA viral infection. Furthermore, we used TRIF-specific siRNA for further experiments to determine whether TRIF can affect cell proliferation and viability upon SCRV infection. As shown in Fig 5F, knockdown of TRIF led to decrease in cell viability using luminescent cell viability assay. When we explored its effect on cell proliferation, knockdown of TRIF resulted in a decline in cell proliferation upon SCRV infection (Fig 5G). Collectively, these data verified that similar to mammals, fish TRIF could mediate the activation of NF-κB and IRF3. In addition, suppression of fish TRIF expression could block IFNs production, exacerbate viral replication, and inhibit cell proliferation and viability.

### miR-15a-5p inhibits antiviral responses by targeting TRIF

**Fig 6:**
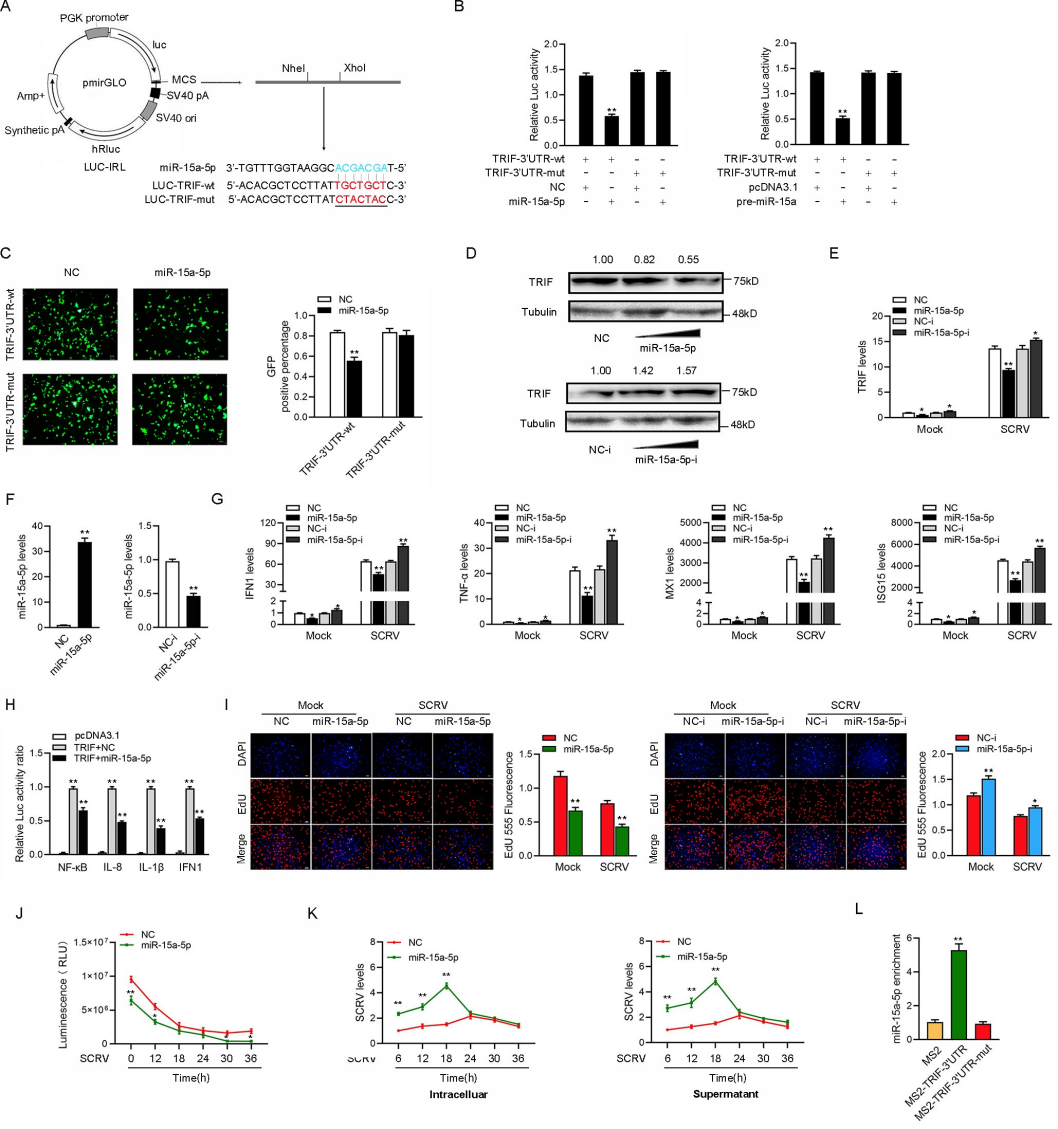
miR-15a-5p inhibits antiviral responses by targeting TRIF. (A) Schematic illustration of TRIF-wt and TRIF-mut luciferase reporter vectors. (B) The relative luciferase activities were detected in EPC cells after cotransfection with TRIF-wt or TRIF-mut and mimics, pre-miR-15a plasmid, or NC. (C) miR-15a-5p downregulated GFP expression. EPC cells were cotransfected with TRIF-3’UTR-wt or TRIF-3’UTR-mut and mimics or NC. The fluorescence intensity and the GFP expression were evaluated by enzyme-labeled instrument. (D and E) Relative protein and mRNA levels of TRIF were evaluated by western blot (D) and qRT-PCR (E) in MIC cells after cotransfected with the miR-15a-5p mimics or inhibitors. (F) The miR-15a-5p expression levels were detected in MIC cells after cotransfection with NC or mimics (left panel) and NC-i or inhibitors (right panel). (G) qRT-PCR assays were performed to determine the expression levels of IFN1, TNF-α, MX1, and ISG15 in cells after cotransfected with NC or mimics and cells after cotransfected with NC-i or inhibitors. (H) The relative luciferase activities were detected in MIC cells after cotransfection with TRIF expression plasmid, phRL-TK Renilla luciferase plasmid, luciferase reporter genes, NC, or mimics. (I and J) Cell proliferation was assessed by EdU assays in MIC cells after cotransfected with NC or mimics (I) and cells after cotransfected with NC-i or inhibitors (J). (K) Effect of miR-15a-5p on cell viability after SCRV infection. MIC cells were transfected with si-Ctrl, si-circ, vector or oe-circ for 24 h, then treated with SCRV for different times. Cell viability assay were measured. (M) miR-15a-5p promote SCRV replication. MIC cells were transfected with si-Ctrl or si-circ and vector or oe-circ plasmid for 24 h, respectively, then infected with SCRV at different times. The qPCR analysis was conducted for intracellular and supernatant SCRV RNA expression. (L) The MS2-RIP assay was executed in MIC cells after transfection with pcDNA3.1-MS2, pcDNA3.1-MS2-TRIF-3’UTR, pcDNA3.1-MS2-TRIF-3’UTR-mut, followed by qPCR to detect the enrichment of miR-15a-5p. Scale bar, 20 μm. All data represented the mean ± SD from three independent triplicated experiments. *, *p* < 0.05; **, *p* < 0.01.

miRNAs could post-transcriptionally regulate the expression of target mRNAs by binding to their 3’UTR. To this end, we predicted the potential target genes of miR-15a-5p using miRNA prediction programs. Among those candidate target genes, the innate antiviral immune related gene TRIF which we are interested contained in it. Thus, we selected TRIF as a target gene of miR-15a-5p for further investigation. We first analyzed the sequence of TRIF 3’UTR and found that miR-15a-5p had a complementary sequence with TRIF 3’UTR (Fig 6A). Then, we constructed the pre-miR-15a overexpression plasmid (Fig 6B). The wild-type and the mutant-type of TRIF 3’UTR were cloned into luciferase reporter vector pmirGLO and then cotransfected with miR-15a-5p mimics or control mimics to determine the interaction between miR-15a-5p and TRIF. The results showed that miR-15a-5p mimics markedly inhibited the luciferase activity when the wild-type 3’UTR was transfected, whereas the mutated form had no response to miR-15a-5p mimics (Fig 6C). We transfected miR-15a-5p mimics and inhibitors into MIC to test whether miR-15a-5p participates in the regulation of TRIF expression. The results from Western blotting and qRT-PCR assays displayed that transfection of miR-15a-5p mimics suppressed the expression levels of TRIF, whereas miR-15a-5p inhibitors markedly enhanced the expression levels of TRIF (Fig 6D).

In addition, we investigated whether miR-15a-5p regulated TRIF at the post-transcriptional level. As shown in Fig 6E, the miR-15a-5p mimics significantly reduced the expression level of TRIF. On the contrary, miR-15a-5p inhibitor significantly increased the expression level of TRIF. Meanwhile, we first investigated the role of miR-15a-5p in regulating IFN1, TNF-α, MX1, and ISG15 to explore the biological function of miR-15a-5p. To this end, we measured the effects of synthetic miR-15a-5p mimics and inhibitors on the expression of miR-15a-5p, and the results indicated that miR-15a-5p mimics enhanced miR-15a-5p expression sharply, whereas miR-15a-5p inhibitors decreased miR-15a-5p expression (Fig 6F). Moreover, the results showed that IFN1, TNF-α, MX1, and ISG15 were significantly decreased by the introduction of miR-15a-5p mimics upon SCRV infection. On the contrary, the inhibition of endogenous miR-15a-5p significantly increased this elevated these gene expressions compared with transfection of control inhibitors (Fig 6G). Next, given that miR-15a-5p targeted TRIF and regulated its expression, we aimed to test whether miR-15a-5p affected TRIF-mediated activation of NF-κB and IRF3. The results from dual-luciferase reporter assays showed that after cotransfection of TRIF overexpression plasmid, miR-15a-5p mimics suppressed the activity of NF-κB, IL-8, IL-1β, and IFN1 luciferase reporters compared with control mimics (Fig 6H). Furthermore, we attempted to investigate whether miR-15a-5p could regulate cell proliferation after SCRV infection. As shown in Fig 6I and 6J, overexpression of miR-15a-5p decreases cell proliferation, whereas the inhibition of miR-15a-5p led to an efficiently increased in cell proliferation. When we investigated the effect of miR-15a-5p on cell viability, we found that overexpression of miR-15a-5p significantly inhibited cell viability upon SCRV infection (Fig 6K). In addition, we examined the effect of miR-15a-5p on SCRV replication to explore the biological significance of miR-15a-5p in SCRV-induced host cells. By detecting the SCRV RNA level, we found that miR-15a-5p significantly promoted SCRV replication before 24h of SCRV infection (Fig 6M). To confirm the direct interaction between the TRIF-3’UTR and the miR-15a-5p, we conducted RIP assays by pcDNA3.1-MS2, pcDNA3.1-MS2-TRIF-3’UTR, and pcDNA3.1-MS2-TRIF-3’UTR-mut. The results from qPCR analysis indicated that miR-15a-5p was efficiently pulled down by pcDNA3.1-MS2-TRIF-3’UTR, but not pcDNA3.1-MS2-TRIF-3’UTR-mut (Fig 6L). Collectively, these data demonstrated that TRIF-3’UTR is able to directly bind to miR-15a-5p, and miR-15a-5p could inhibit antiviral responses and promote the replication of SCRV virus.

### circDtx1 serves as a sponge of miR-15a-5p to enhance TRIF expression

**Fig 7:**
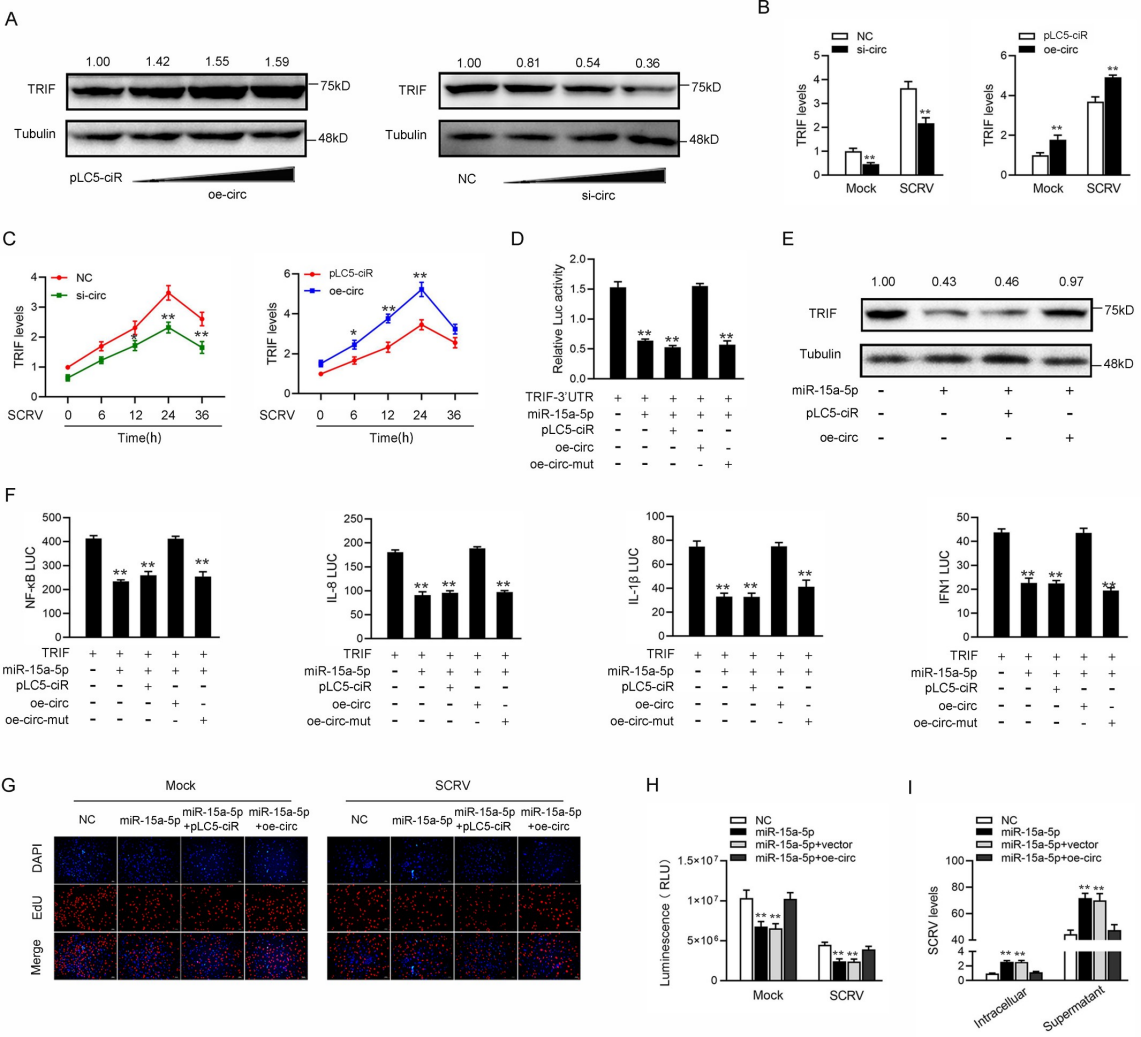
circDtx1 acts as a sponge of miR-15a-5p to enhance TRIF expression. (A and B) Relative mRNA and protein levels of TRIF in MIC or MKC cells after cotransfected with NC, si-circ, vector, or oe-circ by western blot (A) and qPCR assays (B). (C) Relative mRNA levels of TRIF in MIC or MKC cells after cotransfected with si-circ-NC, si-circ, vector, or oe-circ by qPCR assay. (D) The relative luciferase activities were detected in EPC cells after cotransfection with TRIF 3’UTR luciferase reporter vector, NC, mimics, oe-circ or oe-circ-mut. (E) Western blot assays were detected in MKC cells after cotransfection with TRIF overexpression plasmid, NC, mimics, or oe-circ. (F) oe-circ counteracts the negative effect of miR-15a-5p. The relative luciferase activities were detected in MIC cells after cotransfection with TRIF expression plasmid, phRL-TK Renilla luciferase plasmid, luciferase reporters, NC, mimics, or oe-circ. (G) Cell proliferation was assessed by EdU assays in MIC cells after cotransfected with NC, mimics, or oe-circ. (H) Cell viability was assessed by ATP viability assays in MIC cells after cotransfected with NC, mimics, or oe-circ. (I) SCRV RNA expression was assessed by qPCR in MIC cells after cotransfected with NC, mimics, or oe-circ. All data represented the mean ± SD from three independent triplicated experiments. *, *p* < 0.05; **, *p* < 0.01.

Given that circDtx1 could interact with miR-15a-5p and miR-15a-5p targets TRIF and regulates its expression, we tested whether circDtx1 could regulate TRIF. As shown in Fig 7A, overexpression of circDtx1 increased the expression of TRIF protein, while knockdown of circDtx1 significantly reduced the protein level of TRIF. In addition, the qPCR results showed that knockdown of circDtx1 led to reducing the expression levels of TRIF in cells treated with SCRV and Poly (I:C) (left panel of Fig 7B). By contrast, overexpression of circDtx1 increased the expression of TRIF and its mRNA levels (right panel of Fig 7B). Furthermore, as shown in Fig 7C, overexpression of circDtx1 increased the expression levels of TRIF in cells treated with SCRV, whereas knockdown of circDtx1 reduced TRIF expression. Then, we tested whether circDtx1 regulated TRIF expression through miR-15a-5p. To this end, we cotransfected cells with TRIF 3’UTR, together with miR-15a-5p, circDtx1 overexpression plasmid, and circDtx1 mutant plasmid. The results showed that circDtx1 could counteract the inhibitory effect of miR-15a-5p on TRIF 3’UTR (Fig 7D). Strikingly, circDtx1 could also counteract the effect of miR-15a-5p on TRIF expression levels (Fig 7E). These results demonstrated that circDtx1 regulated TRIF expression through miR-15a-5p. Given that miR-15a-5p and TRIF participated in the regulation of NF-κB, IL-8, IL-1β, and IFN1 luciferase reporters, we examined the functional role of circDtx1 in regulating these reporters. The results showed that circDtx1 could counteract the negative effect of miR-15a-5p on the luciferase activities of NF-κB, IL-8, IL-1β, and IFN1 luciferase reporters (Fig 7F). Moreover, we attempted to explore the effect of the circDtx1/miR-15a-5p regulatory loop on cell proliferation. The results indicated that overexpression of circDtx1 could counteract the negative effect of miR-15a-5p on cell proliferation upon SCRV infection (Fig 7G). Meanwhile, we attempted to explore the effect of the circDtx1/miR-15a-5p regulatory loop on cell viability. The results showed that overexpression of circDtx1 could counteract the negative effect of miR-15a-5p on cell viability upon SCRV infection (Fig 7H). In addition, we found that circDtx1 could counteract the promoting effect of miR-15a-5p on SCRV replication (Fig 7I). Collectively, these data demonstrated that circDtx1 served as a ceRNA for miR-15a-5p to regulate TRIF expression.

### ceRNA network that regulates TRIF is widely found in teleost fish

**Fig 8:**
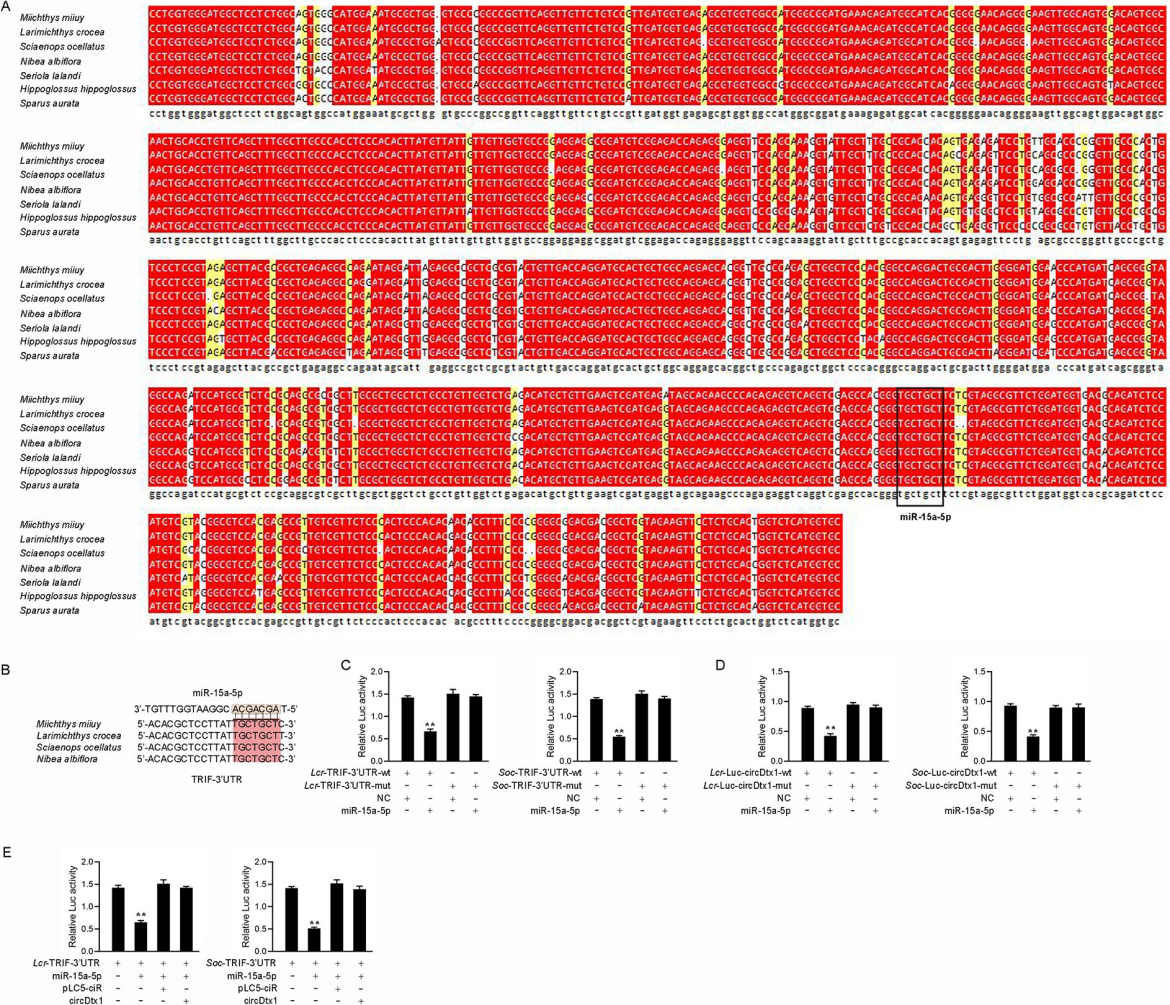
The ceRNA network of regulating TRIF is widely found in teleost fish. (A) Sequence alignment of circDtx1 from various teleost fish species. (B) Sequence alignment of TRIF containing miR-15a-5p binding site among different teleost species. (C) miR-15a-5p regulating the luciferase activity of TRIF-3’UTR is examined in *L*. *croceas* and *S*. *ocellatus*. (D) The relative luciferase activities were detected in EPC cells after cotransfected with *Lc*rcircDtx1-wt or *Lc*rcircDtx1-mut and mimics or NC (left panel) and cells after cotransfected with *Soc*circDtx1-wt or *Soc*circDtx1-mut and mimics or NC (right panel). (E) *Lcr*circDtx1 and *Soc*circDtx1 reduces miR-15a-5p activity. EPC cells were transfected with *Lcr*circDtx1 or *Soc*circDtx1 expression plasmid, mimics, control vector, together with TRIF-3’UTR, the luciferase activity was analyzed and normalized to renilla luciferase activity. All data represented the mean ± SD from three independent triplicated experiments. *, *p* < 0.05.

We performed sequence alignment of circDtx1 from different teleost fish to illustrate the universality of our findings. Interestingly, as shown in Fig 8A, circDtx1 showed high conservation in different fish species. In addition, the binding site of miR-15a-5p in circDtx1 also showed high conservation (Fig 8A). In addition, we analyzed the binding sites of miR-15a-5p and TRIF in other species, and the TRIF showed high conservation at the site of miR-15a-5p in different fish species (Fig 8B). First, we hypothesized that miR-15a-5p might interact with TRIF across different fish species. In verifying this hypothesis, the TRIF 3’UTR sequences of *Larimichthys croceas* and *Sciaenops ocellatus* were cloned into the pmirGLO vector, and their mutated forms with miR-15a-5p binding sites mutated. Significantly, luciferase assays revealed that miR-15a-5p could suppress luciferase activity of the wild form of TRIF 3’UTR luciferase plasmid in both fish species, but it had no effect on mutated forms (Fig 8C). Furthermore, we hypothesized that miR-15a-5p might interact with circDtx1 across different fish species. In verifying this hypothesis, the circDtx1 sequences of *L*. *croceas* and *S*. *ocellatus* were cloned into the pmirGLO vector, and their mutated forms with miR-15a-5p binding sites mutated. Significantly, luciferase assays revealed that miR-15a-5p could suppress luciferase activity of the wild form of circDtx1 luciferase plasmid in both fish species, but it had no effect on mutated forms (Fig 8D). In addition, we conducted luciferase assays to test whether *L*. *crocea* and *S*. *ocellatus* circDtx1 can affect the miR-15a-5p activity and found that both *L*. *crocea* and *S*. *ocellatus* circDtx1 could counteract the inhibitory effect of miR-15a-5p on TRIF 3’UTR (Fig 8E). Collectively, these results showed that circDtx1 could act as endogenous sponge RNA to interact with miR-15a-5p among different teleost fish, which suggested that circDtx1 contains strongly conserved elements among species which is very important for preserving its function.

## Discussion

Viral diseases are one of the most serious threats to the teleost fish. During the viral invasion, the host must identify viral PAMPs in time and evoke appropriate antiviral immune response quickly in order to protect the body from viral invasion. Excessive or weak immune response is not conducive to the survival of organisms under the invasion of the virus; thus, appropriate regulation of immune responses is essential for organisms. In addition, with the deepening of the regulation mechanism of moderate immunity, during the viral invasion, not only one anti-virus immune mechanism is activated, but a variety of anti-virus immune mechanisms work together to resist viral invasion. However, after a long evolution, the virus also has developed numerous strategies to avoid and resist the host’s antiviral immune response. Therefore, different layers of regulatory network mechanisms are necessary to form a more complex system to ensure viral clearance and host preservation. Herein, we reported an interaction network regulating teleost TRIF-mediated antiviral signaling pathways. We found that fish TRIF served as a crucial signaling molecule during SCRV infection, which mediated NF-κB and IRF3 activation and lead to type I IFNs and inflammatory cytokine production. miR-15a-5p can reduce the expression of TRIF and suppress TRIF-mediated antiviral responses, which may help viruses evade host antiviral responses. We further proved that circRNA circDtx1 served as endogenous sponge RNA to interact with miR-15a-5p and facilitate TRIF expression, thereby enhancing the antiviral signaling pathways. Therefore, circDtx1 can counteract the increasing effect of miR-15a-5p on SCRV replication, thereby maintaining the stability of antiviral responses and ensuring appropriate inflammatory responses.

As a virus PRR, TLRs play an irreplaceable role in the antiviral immune response. In teleost, in addition to TLR3, which can transmit antiviral immune signal by TRIF, TLR22 which is unique in the teleost, can also transmit antiviral immune signal by TRIF [44]. TLR22 starts the antiviral immune response by recognizing the dsRNA of the virus, but different from TLR3, it is distributed on the surface of the cell membrane and is primarily responsible for recognizing the extracellular dsRNA virus [44, 45]. Although the only dsRNA can activate the antiviral immune response mediated by TLR3 in theory, many ssRNA viruses can also activate TLR3 and receive antiviral immune response. Most ssRNA viruses can form dsRNA intermediates during replication; thus, they can be recognized by TLR3, and then TRIF transmit signals downstream and activate IRF3, to generating IFN [46, 47]. Recent studies have found that tumor necrosis factor-related factor 3 (TRAF3) and TRAF6 interact with the TRAF6-binding motif present in the N-terminal portion of TRIF through its region. Disruption of the TRAF6-binding motif of TRIF prevents it from connecting to TRAF6, which results in reduced activation of the NF-κB-dependent pathway induced by TRIF without affecting the IFN β promoter [48]. In addition, TANK-binding kinase 1 (TBK1) is connected to the N-terminal region of TRIF, and TRIF linking to TBK1 requires TBK1 kinase activation and phosphorylation of TRIF. Considering that the binding sites of TRAF6 and TBK1 on TRIF are close, TRAF6 may be able to physically prevent the connection of TBK1 and TRIF. Moreover, some reports have shown that IκB kinase I and TRAF-related NF-κB-activating molecule-binding kinase 1 can be used as IRF3 kinases, which are linked not only to IRF3 but also to TRIF [49]. The abovementioned results indicate that TRIF, TRAF6, and TBK1 are linked to activating two different transcription factors, NF-κB and IRF3, respectively [50]. Here, we extended the notion that fish TRIF was involved in the TLR-triggered IFN signaling and provided evidence that similar to mammal TRIF, fish TRIF mediated the activation of NF-κB and IRF3 and leads to type I IFNs production in response to RNA viral infection. Further investigations showed that ncRNAs, including miR-15a-5p and circDtx1, played critical regulatory roles in the TRIF-mediated singling pathway.

In the last decades, research on the regulatory network mechanism of miRNAs is becoming complete and clear in mammals, whereas the research on miRNAs in lower vertebrates (particularly in teleost fish) is also constantly deepening. Particularly, it has become clear that complex miRNA regulation networks exist in teleost fish in regulating innate antiviral immunity. For example, it has been reported that the inducible microRNA-3570 feedback inhibits the RIG-I-dependent innate antiviral immune response to SCRV in teleost fish by targeting mitochondrial anti-viral signaling genes (MAVS/IPS-1) [33]. SCRV-inducible microRNA-210 response modulates antiviral innate immune in miiuy croaker by targeting stimulator of IFN genes (STING/MITA) [32, 51]. In addition, we found a lncRNA MARL in teleost fish, which can target and regulate MAVS mediated antiviral immune response through competitive adsorption of miR-122 [34]. Although many studies have investigated the regulation of antiviral-related genes by small non-coding RNAs in both human and fish, the research on their TRIF regulatory factors is still limited to a variety of proteins; therefore, whether there is a miRNA that can regulate TRIF must be explored. In this study, miRNA miR-15a-5p first proved to be a miRNA-targeting TRIF in miiuy croaker. miR-15a-5p negatively regulates TRIF expression, suppresses TRIF-mediated antiviral responses, and promotes the replication of SCRV. Interestingly, miR-15a-5p promoted SCRV replication only 24 hours before infection (Fig 6K). We think that after the virus invades the host, the host must activate the antiviral immune response to resist the virus invasion, but the activation and function of this response must take a certain time. Therefore, in the late stage of infection, the replication of the virus will be inhibited to a relatively stable level. If it does not reach such a level, either the host will die or the virus will be eliminated. The promotion effect of miR-15a-5p on virus replication must not be lasting. In order to maintain intracellular homeostasis, host cells must regulate this process. The negative regulatory mechanism may be a strategy of RNA virus for their survival by resisting the host antiviral immune response.

**Fig 9:**
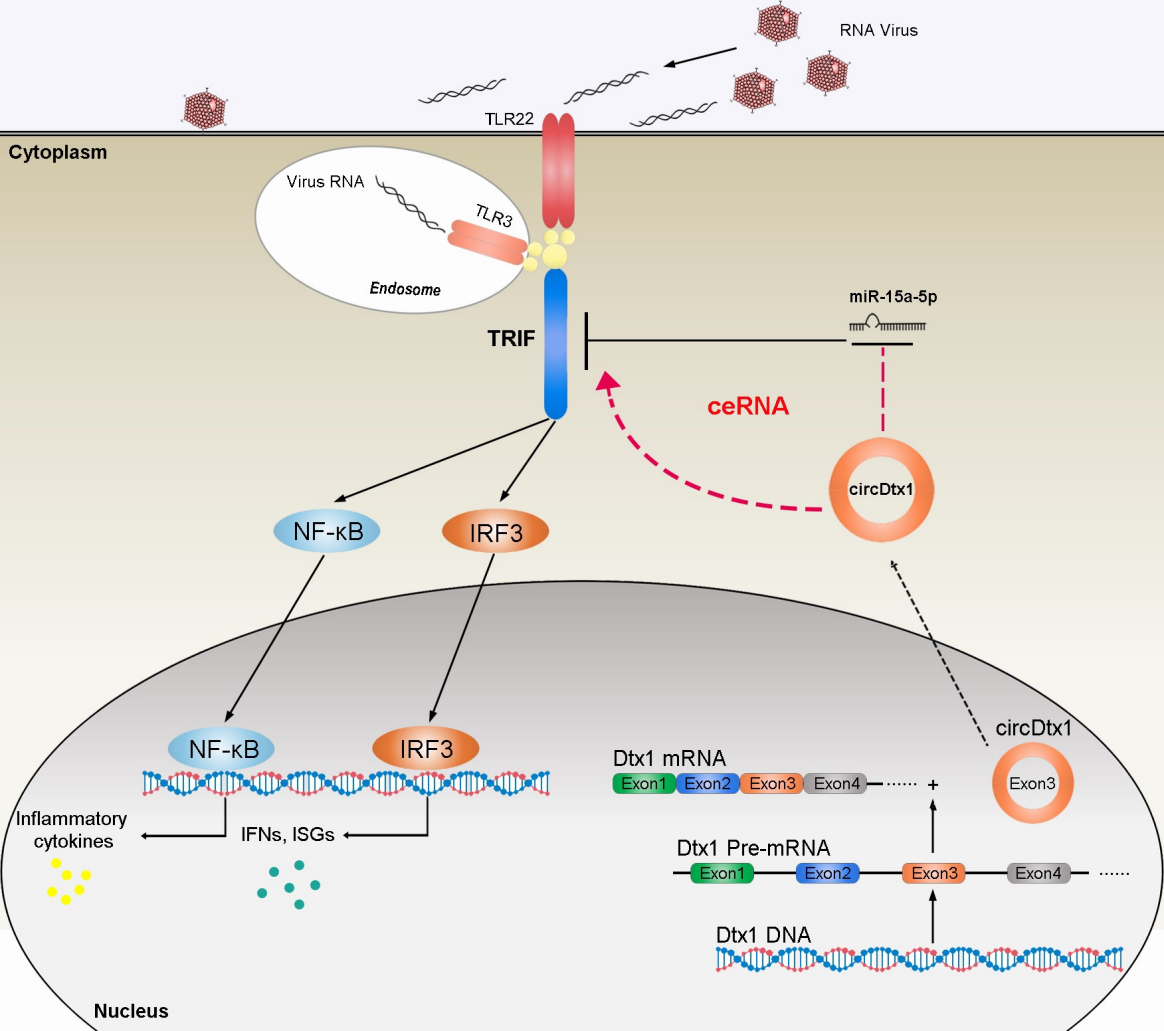
The schematic diagram shows the mechanism underlying circDtx1 as a ceRNA for miR-15a-5p to regulate TRIF expression. miR-15a-5p targets TRIF and represses TRIF-mediated antiviral responses, thereby regulating viral replication. circDtx1 acts as a molecular sponge regulating miR-15a-5p to enhance TRIF expression, thereby maintaining the stability of antiviral responses and ensuring appropriate inflammatory responses.

The ceRNA hypothesis indicates that RNA transcripts including mRNAs, lncRNAs, pseudogenes, and circRNAs can cross-talk and regulate miRNA expression through competing and sharing miRNA response elements (MREs), thereby constructing a new complex post-transcriptional regulatory network and mechanism [52]. CircRNAs can be used as miRNA sponge to regulate mRNA target gene expression. Increasing evidences have shown that the ceRNA mechanism is the primary way for circRNA to play its biological function. For example, circRNA circAGFG1 serves as a sponge of miR-195-5p to promote triple-negative breast cancer progression through regulating cyclin E1 expression [53]. Overexpression of circHLA-C can effectively inhibit lupus nephritis by sponging miR-150 [54]. Additionally, CircRNA-5692 inhibits hepatocellular carcinoma by sponging miR-328-5p to enhance Disabled homolog 2-interacting protein expression [55]. Genes regulated by differentially expressed circular RNAs are involved in a variety of cellular processes, including apoptosis pathways, Janus-activated kinase signal transducers, and activators of transcription pathways, TLRs, and RLRs signaling pathways, and all of which are related to cellular immunity and viral pathogenesis [56]. Therefore, the regulatory mechanism of circRNAs in innate immune responses must be studied. circARF3 alleviates mitophagy-mediated inflammation by targeting miR-103/TRAF3 in mouse adipose tissue [57]; Herein, circDtx1 was predicted to contain miR-15a-5p MREs. Therefore, we hypothesize that circDtx1 may exert antiviral immune responses by binding miR-15a-5p. Moreover, a series of experiments, including biotin-labeled probe pull-down experiment, dual luciferase reporter gene experiment, and RIP experiment, confirmed that circDtx1 can directly interact with miR-15a-5p. Subsequent experiments proved that circDtx1 has a typical ceRNA mechanism. It can be used as a sponge to absorb miR-15a-5p, which indirectly enhanced the expression of TRIF, and promoted the antiviral immune responses. Our current research results show that circDtx1 can indirectly target TRIF through miRNA to promote the production of antiviral genes, thereby resisting viral invasion and replication (Fig 9). This result further proves that circRNAs participate in the regulation of immune responses as a new immunomodulatory molecule.

In this study, we found a circRNA, circDtx1, which was involved in the regulation of innate antiviral immune responses in teleost fish. Among them, miR-15a-5p was also a newly discovered miRNA that could negatively regulate TRIF mediated antiviral immune responses and indirectly promote viral replication. On the contrary, circDtx1 played a positive role in TRIF-mediated antiviral responses. We confirmed that the mechanism of the two ncRNAs played a role in regulating the innate antiviral immune responses. circDtx1 could be used as the ceRNA of miR-15a-5p to reduce its inhibitory effect on TRIF expression, thereby inhibiting the replication of the virus. In addition, we also found that the structure and function of circDtx1 were highly conserved in different teleost fish. In summary, our research revealed that circRNAs were involved in the host-virus interaction mechanism, which provided new insights into the role of circular RNA in host antiviral immunity.

## Methods

### Ethics statement

All animal experimental procedures were performed in accordance with the National Institutes of Health’s Guide for the Care and Use of Laboratory Animals, and the experimental protocols were approved by the Research Ethics Committee of Shanghai Ocean University (No. SHOU-DW-2018-047).

### Sample and challenge

Miiuy croaker (∼ 50 g) was obtained from Zhoushan Fisheries Research Institute, Zhejiang Province, China. Fish was acclimated in aerated seawater tanks at 25°C for six weeks before experiments. Experimental procedures and SCRV infect were performed as described [34].

### Sequencing analysis and circRNAs identification

The spleen tissues from three healthy fishes and three SCRV-challenged fishes were separated and total RNAs were extracted for the construction of the cDNA library. Afterward, the cDNA libraries were sequenced using Illumina HiSeq 2500 platform. The sequencing data have been deposited in the Sequence Read Archive (SRA) at the National Center for Biotechnology Information (NCBI) under accession number PRJNA685924. Clean reads were aligned against the miiuy croaker reference genome using the mapping program TopHat2 [58]. The unmapped reads were extracted and further aligned with miiuy croaker reference sequence by TopHat-fusion software [59]. The junction reads with noncolinear ordering alignment on the same chromosome were regarded as candidate back-spliced junction reads. The back-spliced junction reads were used for the identification of circRNAs by CIRI software [60].

### Cell culture and treatment

*M*. *miiuy* spleen cells (MSpC), *M*. *miiuy* kidney cells (MKC), *M*. *miiuy* muscle cells (MMC), *M*. *miiuy* brain cells (MBrC), and *M*. *miiuy* intestine cells (MIC) were cultured in L-15 medium (HyClone) supplemented with 15% fetal bovine serum (FBS; Gibco), 100 U/ml penicillin, and 100μg/ml streptomycin at 26°C. And the above five cell lines were prepared from the corresponding tissues of the miiuy croaker, and the specific preparation process was as described previously [61]. Fish Epithelioma papulosum cyprini cells (EPC) were maintained in medium 199 (Invitrogen) supplemented with 10% FBS, 100 U/ml penicillin, and 100 mg/ml streptomycin at 28°C in 5% CO2. For stimulation experiments, MKC and MIC cells were challenged with SCRV at a multiplicity of infection (MOI) of 5 and harvested at different times for RNA extraction. SCRV virus was isolated as described [62], and the replication of SCRV was detected by qPCR.

### Plasmids construction

To construct the TRIF 3’UTR reporter vector, the 3’UTR region of *M*. *miiuy* TRIF gene, as well as *L*. *crocea* and *S*. *ocellatus* TRIF 3’UTR, were amplified using PCR and cloned into pmirGLO luciferase reporter vector (Promega). To construct the TRIF expression plasmid of *M*. *miiuy*, the TRIF cDNA was amplified by specific primer pairs and cloned into pcDNA3.1 (Invitrogen) vector. Meanwhile, the TRIF 3’UTR sequences of *M*. *miiuy* was inserted into mVenus-C1 vector (Invitrogen), which included the sequence of enhanced GFP. To construct circDtx1 overexpression vector, the full-length circDtx1 cDNA was amplified by specific primer pairs and cloned into pLC5-ciR vector (Geneseed Biotech), which contained a front and back circular frame to promote RNA circularization. Also, the circDtx1 overexpression vectors of *L*. *crocea* and *S*. *ocellatus* were constructed by synthesizing the full-length circDtx1 cDNA of *L*. *crocea* and *S*. *ocellatus*, respectively. The empty vector with no circDtx1 sequence was used as negative control. The mutated forms with point mutations in the miR-15a-5p binding site were synthesized using Mut Express II Fast Mutagenesis Kit V2 with specific primers. A miR-15a-5p sensor was created by inserting two consecutive miR-15a-5p complementary sequences into the psiCHECK vector (Promega). The correct construction of the plasmids was verified by Sanger sequencing and extracted through EndoFree Plasmid DNA Miniprep Kit (Tiangen Biotech). To build pLC5-ciR-MS2, the MS2 fragment was inserted into the pLC5-ciR vector, and then the MS2 sequence was inserted into any position in the circDtx1 sequence in the pLC5-ciR-Dtx1 vector, except for the binding site of miR-15a-5p. To build pcDNA3.1-MS2, the MS2-12X fragment was inserted into the pcDNA3.1 vector, and then the TRIF-3’UTR was amplified and cloned into pcDNA3.1-MS2. The mutated forms with point mutations in the miR-15a-5p binding site were synthesized using Mut Express II Fast Mutagenesis Kit V2 with specific primers (S1 Table).

### RNA oligoribonucleotides

The miR-15a-5p mimics are synthetic double-stranded RNAs (dsRNAs) with stimulating naturally occurring mature miRNAs. The miR-15a-5p mimics sequence was, 5’-UAGCAGCACGGAAUGGUUUGU-3’. The miR-15a-5p mimics mutant sequence was 5’-UGUAGUAGCGGAAUGGUUUGU-3’. The negative control mimics sequence was 5’-UUCUCCGAACGUGUCACGUTT-3’. miRNA inhibitors are synthetic single-stranded RNAs (ssRNAs) that sequester intracellular miRNAs and block their activity in the RNA interfering pathway. The miR-15a-5p inhibitors sequence was 5’-ACAAACCAUUCCGUGCUGCUA-3’. The negative control inhibitor sequence was 5’-CAGUACUUUUGUGUAGUACAA-3’. The RNA interference for circDtx1 are as follows: si-circDtx1-1 sequence was 5’- GGUCUCAUGGUGCCCCUGGTT-3’; si-circDtx1-2 sequence was 5’-ACUGGUCUCAUGGUGCCCCTT-3’. The scrambled control RNA sequences were 5’- GGUCUCAUGGUGCAAUCAATT-3’. The RNA interference for TRIF is as follows: si-TRIF sequence was 5’-GAGACAACUACCUUGCUAGTT-3’. The negative control mimics sequence was 5’-UUCUCCGAACGUGUCACGUTT-3’.

### Cell transfection

Transient transfection of cells with miRNA mimic, miRNA inhibitor, or siRNA was performed in 24-well plates using Lipofectamine RNAiMAX (Invitrogen), and cells were transfected with DNA plasmids were performed using Lipofectamine 3000 (Invitrogen) according to the manufacturer’s instructions. For functional analyses, the overexpression plasmid (500 ng per well) or control vector (500 ng per well) and miRNA mimics (100 nM), miRNA inhibitor (100nM), or siRNA (100nM) were transfected into cells in culture medium and then harvested for further detection. For luciferase experiments, miRNA mimics (100 nM) or miRNA inhibitor (100nM) and pmirGLO (500 ng per well) containing the wild or mutated plasmid of TRIF 3’UTR were transfected into cells.

### RNA extract and quantitative real-time PCR

For the isolation and purification of both cytoplasmic and nuclear RNA from MIC cells, the Cytoplasmic & Nuclear RNA Purification Kit has been used according to the manufacturer’s instructions (Norgen Biotek). Total RNA was isolated with TRIzol Reagent (Invitrogen) and the cDNA was synthesized using the FastQuant RT Kit (Tiangen) which includes DNase treatment of RNA to eliminate genomic contamination. The expression patterns of each gene were performed by using SYBR Premix Ex Taq (Takara). The small RNA was extracted by using miRcute miRNA Isolation Kit (Tiangen), and miRcute miRNA FirstStrand cDNA Synthesis Kit (Tiangen) was applied to reverse transcription of miRNAs. The expression analysis of miR-15a-5p was executed by using the miRcute miRNA qPCR Detection Kit (Tiangen). Real-time PCR was performed in an Applied Biosystems QuantStudio 3 (Thermo Fisher Scientific). GAPDH and 5.8S rRNA were employed as endogenous controls for mRNA and miRNA, respectively. Primer sequences are displayed in S1 Table.

### Luciferase report assay

The wild-type of circDtx1 and the mutant devoid of the miR-15a-5p binding site were cotransfected with miR-15a-5p mimics into EPC cells. At 48 h post-transfection, reporter luciferase activities were measured using the dual-luciferase reporter assay system (Promega). To determine the functional regulation of circDtx1, cells were cotransfected TRIF overexpression plasmid or circDtx1 overexpression plasmid, together with NF-κB, IL-8, IL-1β, and IFN1 luciferase reporter gene plasmids, phRL-TK Renilla luciferase plasmid, either miR-15a-5p mimics or negative controls. At 48 h post-transfection, the cells were lysed for reporter activity using the dual-luciferase reporter assay system (Promega). The miR-15a-5p sensor was cotransfected with miR-15a-5p mimics or circDtx1 overexpression plasmid. At 48 h post-transfection, the cells were lysed for reporter activity. All the luciferase activity values were achieved against the renilla luciferase control. Transfection of each construct was performed in triplicate in each assay. Ratios of renilla luciferase readings to firefly luciferase readings were taken for each experiment, and triplicates were averaged.

### Western blotting

Cellular lysates were generated by using 1×SDS-PAGE loading buffer. Proteins were extracted from cells and measured with the BCA Protein Assay kit (Vazyme), then subjected to SDS-PAGE (8%) gel and transferred to PVDF (Millipore) membranes by semidry blotting (Bio-Rad Trans Blot Turbo System). The membranes were blocked with 5% BSA. Protein was blotted with different antibodies. The antibody against TRIF was diluted at 1: 500 (Abcam); anti-Flag and anti-Tubulin monoclonal antibody were diluted at 1: 2,000 (Sigma); and HRP-conjugated anti-rabbit IgG or anti-mouse IgG (Abbkine) at 1: 5,000. The results were representative of three independent experiments. The immunoreactive proteins were detected by using WesternBright ECL (Advansta). The digital imaging was performed with a cold CCD camera.

### RNase R treatment

The RNAs (10 μg) from MIC and MKC cells were treated with RNase R (3 U/μg, Epicenter) and incubated for 30 min at 37°C. Then, the treated RNAs were reverse transcribed with a divergent primer or convergent primer and detected by qPCR and RT-PCR assay followed by nucleic acid electrophoresis.

### Nucleic acid electrophoresis

The cDNA and gDNA PCR products were investigated using 2% agarose gel electrophoresis with TAE running buffer. DNA was separated by electrophoresis at 100 V for 30 min. The DNA marker was Super DNA Marker (100–10,000 bp) (CWBIO). The bands were examined by UV irradiation.

### RNA pulldown assay

circDtx1 and circDtx1-mut with miR-15a-5p binding sites mutated were transcribed *in vitro*. The two transcripts were biotin-labeled with the T7 RNA polymerase and Biotin RNA Labeling Mix (Roche), treated with RNase-free DNase I, and purified with an RNeasy Mini Kit (Qiagen). The whole-cell lysates from MIC cells (~1.0 × 10^7^) were incubated with purified biotinylated transcripts for 1 h at 25°C. The complexes were isolated by streptavidin agarose beads (Invitrogen). RNA was extracted from the remaining beads and qPCR was used to evaluate the expression levels of miRNAs.

To conduct pulldown assay with biotinylated miRNA, MIC cells were harvested at 48 h after transfection, then incubated on ice for 30 min in lysis buffer (20 mM Tris, pH 7.5, 200mM NaCl, 2.5 mM MgCl_2_, 1mM DTT, 60 U/ml Superbase-In, 0.05% Igepal, protease inhibitors). The lysates were precleared by centrifugation for 5 min, and 50 μl of the sample was aliquoted for input. The remaining lysates were incubated with M-280 streptavidin magnetic beads (Sigma). To prevent non-specific binding of RNA and protein complexes, the beads were coated with RNase-free BSA and yeast tRNA (Sigma). The beads were incubated for 4 h at 4°C, washed twice with ice-cold lysis buffer, three times with the low salt buffer (0.1% SDS, 1% Triton X-100, 2 mM EDTA, 20 mM Tris-HCl pH 8.0 and 150 mM NaCl) and once with the high salt buffer (0.1% SDS, 1% Triton X-100, 2 mM EDTA, 20 mM Tris-HCl pH 8.0 and 500 mM NaCl). RNA was extracted from the remaining beads with TRIzol Reagent (Invitrogen) and evaluated by qPCR.

### RNA immunoprecipitation assay (RIP)

RIP experiments were performed by using the Magna RIP RNA-Binding Protein Immunoprecipitation Kit (Millipore) following the manufacturer’s protocol. The Ago-RIP assay was conducted in MIC cells (~2.0×10^7^) transfected Ago2-flag or pcDNA3.1-flag and miR-15a-5p mimics or control mimics. After 48 h transfection, the cells were extract was incubated with magnetic beads conjugated with IgG and anti-Flag antibody (Sigma). RNA was extracted from the remaining beads and qPCR was used to evaluate the expression levels of circDtx1.

The MS2-RIP assay was also conducted in MIC cells (~2.0 × 10^7^) transfected with pLC5-ciR-MS2, pLC5-ciR-MS2-circDtx1, pLC5-ciR-MS2-circDtx1-mut, or pMS2-GFP (Addgene). To construct plasmids that could produce circDtx1 identified by the MS2 protein, an MS2 fragment was cloned into pLC5-ciR, pLC5-ciR-circDtx1, and mutated type of circDtx1 plasmid. Furthermore, a GFP and MS2 gene fusion expression plasmid was also constructed to produce a GFP-MS2 fusion protein that could bind with the MS2 fragment and be identified using an anti-GFP antibody (Abcam). After 48 h transfection, the MIC cells were used in RIP assays via the Magna RIP RNA-Binding Protein Immunoprecipitation Kit (Millipore) and an anti-GFP antibody following the manufacturer’s protocol. RNA was extracted from the remaining beads and qPCR was used to evaluate the expression levels of miRNAs.

### Cell viability and proliferation assay

Cell viability was measured 48 h after transfection in SCRV-treated MIC with Celltiter-Glo Luminescent Cell Viability assays (Promega) according to the manufacturer’s instructions. The EdU assay was performed to assess the proliferation of cells by using BeyoClick EdU cell Proliferation Kit with Alexa Fluor 555 (Beyotime) following the manufacturer’s instructions. The EdU cell lines were photographed and counted under a Leica DMiL8_fluorescence microscope and evaluated by Thermo Scientific Varioskan LUX. These experiments were repeated three times.

### Statistical analysis

Data are expressed as the mean ± SD from at least three independent triplicated experiments. Student’s t-test was used to evaluate the data. The relative gene expression data was acquired using the 2 ^-ΔΔCT^ method, and comparisons between groups were analyzed by one-way analysis of variance (ANOVA) followed by Duncan’s multiple comparison tests [49]. A value of *p* < 0.05 was considered significant.

## Supporting information

S1 Table PCR primer information in this study. (DOCX)

S1 Fig Full length sequence of Dtx1 gene of miiuy croaker. (TIF)
