# Correction: Circular RNA circDtx1 regulates IRF3-mediated antiviral immune responses through suppression of miR-15a-5p-dependent TRIF downregulation in teleost fish

**DOI:** 10.1371/journal.ppat.1013058

**Published:** 2025-04-07

**Authors:** The PLOS Pathogens Editors

Following publication of this article [1], questions were raised about similarity between this work and other studies by the same group, including about similarities in standard deviations and the relative positioning of data points in some charts and graphs. An investigation by Shanghai Ocean University found that the data reported in [1] are reliable and valid, and advised that similarities across studies may reflect the standards and thresholds applied by the researchers when selecting specific molecules to investigate following a large-scale screen to identify functional molecules.

The *PLOS Pathogens* Editors consider the above issues resolved, and issue this Correction to address additional items as follows:

Contrary to the Data Availability statement accompanying this article [1], the complete underlying data were not made available at the time of publication. The available individual-level quantitative data are provided in S1 File with this notice. Raw qPCR data are no longer available. The available underlying image data are provided in S2 File with this notice.

Editorial assessment of the underlying blot images identified that published western blot panels in Figs 6D and 7E show tubulin loading control bands taken from different lanes compared to the corresponding target protein lanes. Additionally, the left-hand and right-hand TRIF panels in Fig 7A appear to be taken from lanes 1-4 and 7-10 of the same blot, while the tubulin panels in Fig 7A appear to be taken from two separate underlying blots. The authors stated that the control and experimental blots were generated from different gels, and in some instances samples were not loaded in the same lane positions. An inspection of laboratory research records by Shanghai Ocean University determined that the blot results are correctly reported in [1].

The *PLOS Pathogens* Editors note that several sentences in the Abstract, Author Summary, and Results reuse text from the authors’ prior articles. Specifically, parts of the text in the Abstract and in the Results subsection “circDtx1 functions as a miRNA sponge of miR-15a-5p” overlap with text in [2,3], and parts of the text in the Author Summary and in the Results subsection “Fish TRIF enhance antiviral responses upon SCRV infection” overlap with text in [4].

The ethical approval document issued by the Research Ethics Committee of Shanghai Ocean University has been provided for editorial review, and a representative of Shanghai Ocean University has confirmed that the procedures reported in [1] are in accordance with this ethical approval and conducted within its period of validity.

## Supporting information

S1 File Individual-level quantitative data underlying charts and graphs.
(ZIP)

S2 File Underlying image data for Figures 1, 2, 4, 5, 6, and 7.
(ZIP)
