# Supplementary material for: Circular RNA circDtx1 regulates IRF3-mediated antiviral immune responses through suppression of miR-15a-5p-dependent TRIF downregulation in teleost fish
Source: PLoS Pathog. 2021 Mar 18;17(3):e1009438. doi: 10.1371/journal.ppat.1009438 (PMC8009406; doi:10.1371/journal.ppat.1009438)
Supplement: S1 Table — (DOCX) [file ppat.1009438.s001.docx]

**Table S1** PCR primer information in this study.

| Primer | Sequences (5’-3’) |
| --- | --- |
| TRIF-qRT-F | TGTCCCAATGTGCCACCA |
| TRIF-qRT-R | TCACCTCCACCCAATCCC |
| TNF-α-qRT-F | GTTTGCTTGGTACTGGAATGG |
| TNF-α-qRT-R | TGTGGGATGATGATCTGGTTG |
| IFN1-qRT-F | TACGATGGCTAATAACTCC |
| IFN1-qRT-R | CATTGACAAAGTGCTCCA |
| MX1-qRT-F | GCTGCTTGTTTACTCCCA |
| MX1-qRT-R | ACCTGCATCATCTCCCTC |
| ISG15-qRT-F | TGAACGGACAGAAGACGC |
| ISG15-qRT-R | TGAGGAATACCTGCATGG |
| SCRV-qRT-F | GGGCTGGATGATAGACGATTG |
| SCRV-qRT-R | TGGCGGAGGTGCTTGATATGG |
| circDtx1-divergent-F | CGTAGGCGTTCTGGAT |
| circDtx1-divergent-R | AGGGGCACCATGAGAC |
| circDtx1-convergent-F | AGGACTGCGACTTGGG |
| circDtx1-convergent-R | TGGGCTTCTGCTATCTC |
| miR-15a-5p-qRT-F | CAGTAGCAGCACGGAATG |
| miR-15a-5p-qRT-R | CCAGTTTTTTTTTTTTTTTACAAACCA |
| miR-455-1-3p-qRT-F | TGCAGTCCATGGGCAT |
| miR-455-1-3p -qRT-R | GGTCCAGTTTTTTTTTTTTTTTGTATATG |
| miR-133-5p-qRT-F | CCGCAGTCTGGTGTC |
| miR-133-5p-qRT-R | GTCCAGTTTTTTTTTTTTTTTGCG |
| miR-63-5p-qRT-F | GCATGGGGACGGGAAG |
| miR-63-5p-qRT-R | GTCCAGTTTTTTTTTTTTTTTCCATAC |
| miR-98-3p-qRT-F | GCAGGGACAGAAACATGG |
| miR-98-3p-qRT-R | GGTCCAGTTTTTTTTTTTTTTTACAG |
| 5.8S rRNA-qRT-F | AACTCTTAGCGGTGGATCA |
| 5.8S rRNA-qRT-R | GTTTTTTTTTTTTTTTGCCGAGTG |
| GAPDH-qRT-F | ACCTTCACTCCTCCATCTT |
| GAPDH-qRT-R | AGGTCACAGACACGGTTG |
| circDtx1-F | CGGAATTCTAATACTTTCAGCCCTGGTGGGATGGCTCCTC |
| circDtx1-R | CGGGATCCAGTTGTTCTTACCACCATGAGACCAGTGCAG |
| circDtx1-pmirGLO-F | CGAGCTCCCCTGGTGGGATGGCTCCTC |
| circDtx1-pmirGLO-R | TGCTCTAGACACCATGAGACCAGTGCAG |
| circDtx1-pmirGLO-mut-F | ACGGGCTACTATTCTCGTAGGCGTTCTGGATGG |
| circDtx1-pmirGLO-mut-R | CGAGAATAGTAGCCCGTGGCTCGACCTGACCT |
| circDtx1-mVenus-F | TCAGATCTCGAGCTCAAGCTTCCCTGGTGGGATGGCTCC |
| circDtx1-mVenus-R | CGGGCCCGCGGTACCGTCGACGCACCATGAGACCAGTGCAGA |
| circDtx1-mVenus-mut-F | ACGGGCTACTATTCTCGTAGGCGTTCTGGATGG |
| circDtx1-mVenus-mut-R | CGAGAATAGTAGCCCGTGGCTCGACCTGACCT |
| miR-15a-5p sensor-F | TCGAGATCGTCGTGCCTTACCAAACAATCGTCGTGCCTTACCAAACAGC |
| miR-15a-5p sensor-R | GGCCGCTGTTTGGTAAGGCACGACGATTGTTTGGTAAGGCACGACGATC |
| Ago2-Flag-F | CCCAAGCTTGACAAAATGTATTCCTCTGC |
| Ago2-Flag-R | CGCGGATCCTTTCATCAGTGGGGTCTC |
| circDtx1-T7-F | TAATACGACTCACTATAGGGGGCTCTGCCTGTTGGTC |
| circDtx1-T7-R | AATACCTTTGCTGGAACCTC |
| pLC5-MS2-circDtx1-F | ACATGAGGATCACCCATGTCTGCAGTAGAGCTTACGCCGCTGAGAG |
| pLC5-MS2-circDtx1-R | CATGGGTGATCCTCATGTTTTCTAGCGGAGGGACAGTGGGCAA |
| TRIF-HindIII-F | GACGATGACGACAAGAAGCTTATGAGCCGCGAGGGAGAA |
| TRIF-EcoRI-R | TGATGGATATCTGCAGAATTCAAACAAGCATACACACGAGTTATACAGC |
| TRIF-3’UTR-F | CTAGCTAGCGCTGATTGTAGAAATTGTGAGAGGT |
| TRIF-3’UTR-R | TGCTCTAGAAAACAAGCATACACACGAGT |
| TRIF-3’UTR-mVenus-F | CCCAAGCTTGCTGATTGTAGAAATTGTGAGAGGT |
| TRIF-3’UTR-mVenus-R | CGCGGATCCAAACAAGCATACACACGAGT |
| TRIF-3’UTR-mut-F | GCTCCTTATCTACTACCATTGCTCTTCCCAGCATCTTT |
| TRIF-3’UTR-mut-R | GGTAGTAGATAAGGAGCGTGTAACAGCATTGC |
| TRIF-3’UTR-MS2-F | ACTATAGGGAGACCCAAGCTTATGTAAAAATATGATGACATATGGTTAGAAT |
| TRIF-3’UTR-MS2-R | GCGGCCGTTACTAGTGGATCCAAACAAGCATACACACGAGTTATACAGC |
| *Lcr*TRIF-3’UTR-F | CTAGCTAGCGCTGATATGGTTAGAATACGATGAT |
| *Lcr*TRIF-3’UTR-R | TGCTCTAGACACAGGAGATGGGAAA |
| *Lcr*TRIF-3’UTR-mut-F | GCTCCTTATCTACTACCATTGCTCTTCCCAGCATCTTT |
| *Lcr*TRIF-3’UTR-mut-R | GGTAGTAGATAAGGAGCGTGTAACAGCATTGC |
| *Soc*TRIF-3’UTR-F | CTAGCTAGCGCTGATTTGTAGAAATTGTGAGCGA |
| *Soc*TRIF-3’UTR-R | TGCTCTAGAAAAGCAACCTGCCTGA |
| *Soc*TRIF-3’UTR-mut-F | GCTCCTTATCTACTACCATTGCTCTTCCCAGCATCTTT |
| *Soc*TRIF-3’UTR-mut-R | GGTAGTAGATAAGGAGCGTGTAACAGCATTGC |
| *Lcr*circDtx1-F | CGGAATTCTAATACTTTCAGCCCTGGTGGGATGGCTCCTC |
| *Lcr*circDtx1-R | CGGGATCCAGTTGTTCTTACCACCATGAGACCGGTGCAG |
| *Lcr*circDtx1-pmirGLO-F | CGAGCTCCCCTGGTGGGATGGCTCCTC |
| *Lcr*circDtx1-pmirGLO-R | TGCTCTAGACACCATGAGACCGGTGCAG |
| *Lcr*circDtx1-pmirGLO-mut-F | ACGGGCTACTATTCTCGTAGGCGTTCTGGATGG |
| *Lcr*circDtx1-pmirGLO-mut-R | CGAGAATAGTAGCCCGTGGCTCGACCTGACCT |
| *Soc*circDtx1-F | CGGAATTCTAATACTTTCAGCCCTGGTGGGATGGCTCCTC |
| *Soc*circDtx1-R | CGGGATCCAGTTGTTCTTACCACCATGAGACCAGTGCAG |
| *Soc*circDtx1-pmirGLO-F | CGAGCTCCCCTGGTGGGATGGCTCCTC |
| *Soc*circDtx1-pmirGLO-R | TGCTCTAGACACCATGAGACCAGTGCAG |
| *Soc*circDtx1-pmirGLO-mut-F | ACGGGCTACTATTCTCGTAGGCGTTCTGGATGG |
| *Soc*circDtx1-pmirGLO-mut-R | CGAGAATAGTAGCCCGTGGCTCGACCTGACCT |
