# Supplementary figures and images for: Circular RNA circDtx1 regulates IRF3-mediated antiviral immune responses through suppression of miR-15a-5p-dependent TRIF downregulation in teleost fish
Source: PLoS Pathog. 2021 Mar 18;17(3):e1009438. doi: 10.1371/journal.ppat.1009438 (PMC8009406; doi:10.1371/journal.ppat.1009438)

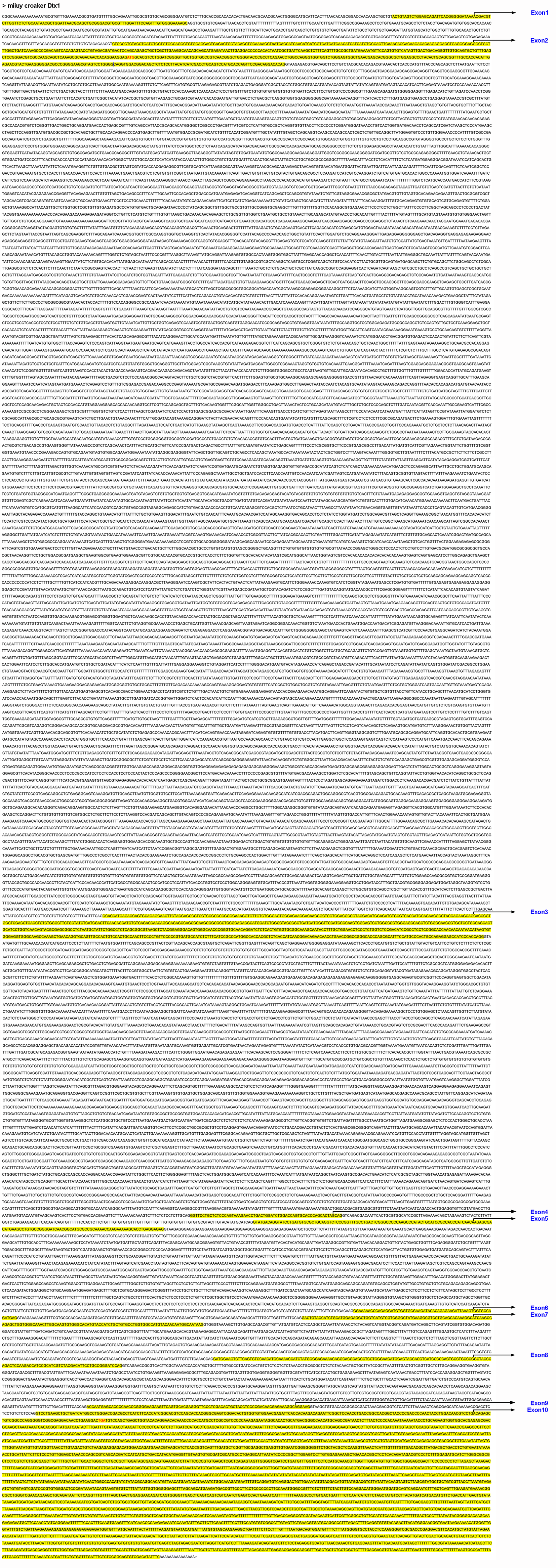

Supplement: S1 Fig — (TIF) [file ppat.1009438.s002.tif]
