# Supplementary figures and images for: Correction: Circular RNA circDtx1 regulates IRF3-mediated antiviral immune responses through suppression of miR-15a-5p-dependent TRIF downregulation in teleost fish (part 2 of 2)
Source: PLoS Pathog. 2025 Apr 7;21(4):e1013058. doi: 10.1371/journal.ppat.1013058 (PMC11975111; doi:10.1371/journal.ppat.1013058)

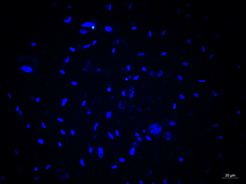

Supplement: S2 File — Underlying image data for Figures 1, 2, 4, 5, 6, and 7. (ZIP) [file ppat.1013058.s002.zip › S2 File/Original image-DOI 10.1371.journal.ppat.1009438/Figure7-Detailed raw data/7G/Fig7G-DAPI-8.tif]

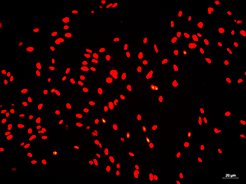

Supplement: S2 File — Underlying image data for Figures 1, 2, 4, 5, 6, and 7. (ZIP) [file ppat.1013058.s002.zip › S2 File/Original image-DOI 10.1371.journal.ppat.1009438/Figure7-Detailed raw data/7G/Fig7G-EdU-1.tif]

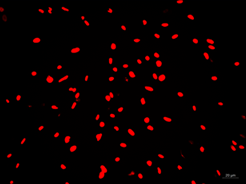

Supplement: S2 File — Underlying image data for Figures 1, 2, 4, 5, 6, and 7. (ZIP) [file ppat.1013058.s002.zip › S2 File/Original image-DOI 10.1371.journal.ppat.1009438/Figure7-Detailed raw data/7G/Fig7G-EdU-2.tif]

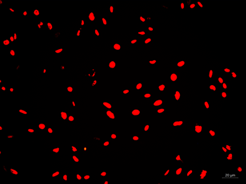

Supplement: S2 File — Underlying image data for Figures 1, 2, 4, 5, 6, and 7. (ZIP) [file ppat.1013058.s002.zip › S2 File/Original image-DOI 10.1371.journal.ppat.1009438/Figure7-Detailed raw data/7G/Fig7G-EdU-3.tif]

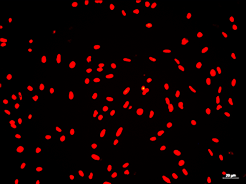

Supplement: S2 File — Underlying image data for Figures 1, 2, 4, 5, 6, and 7. (ZIP) [file ppat.1013058.s002.zip › S2 File/Original image-DOI 10.1371.journal.ppat.1009438/Figure7-Detailed raw data/7G/Fig7G-EdU-4.tif]

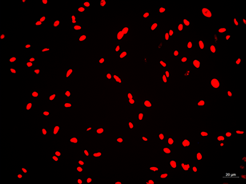

Supplement: S2 File — Underlying image data for Figures 1, 2, 4, 5, 6, and 7. (ZIP) [file ppat.1013058.s002.zip › S2 File/Original image-DOI 10.1371.journal.ppat.1009438/Figure7-Detailed raw data/7G/Fig7G-EdU-5.tif]

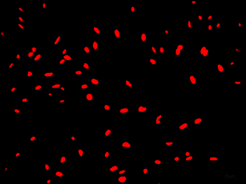

Supplement: S2 File — Underlying image data for Figures 1, 2, 4, 5, 6, and 7. (ZIP) [file ppat.1013058.s002.zip › S2 File/Original image-DOI 10.1371.journal.ppat.1009438/Figure7-Detailed raw data/7G/Fig7G-EdU-6.tif]

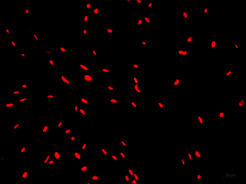

Supplement: S2 File — Underlying image data for Figures 1, 2, 4, 5, 6, and 7. (ZIP) [file ppat.1013058.s002.zip › S2 File/Original image-DOI 10.1371.journal.ppat.1009438/Figure7-Detailed raw data/7G/Fig7G-EdU-7.tif]

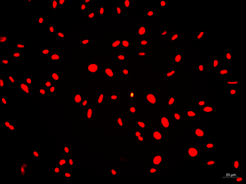

Supplement: S2 File — Underlying image data for Figures 1, 2, 4, 5, 6, and 7. (ZIP) [file ppat.1013058.s002.zip › S2 File/Original image-DOI 10.1371.journal.ppat.1009438/Figure7-Detailed raw data/7G/Fig7G-EdU-8.tif]

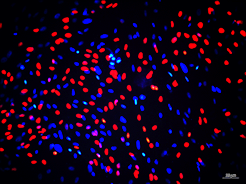

Supplement: S2 File — Underlying image data for Figures 1, 2, 4, 5, 6, and 7. (ZIP) [file ppat.1013058.s002.zip › S2 File/Original image-DOI 10.1371.journal.ppat.1009438/Figure7-Detailed raw data/7G/Fig7G-Merge-1.tif]

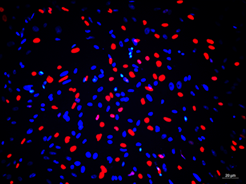

Supplement: S2 File — Underlying image data for Figures 1, 2, 4, 5, 6, and 7. (ZIP) [file ppat.1013058.s002.zip › S2 File/Original image-DOI 10.1371.journal.ppat.1009438/Figure7-Detailed raw data/7G/Fig7G-Merge-2.tif]

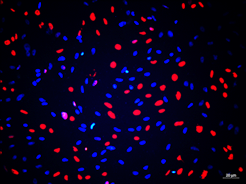

Supplement: S2 File — Underlying image data for Figures 1, 2, 4, 5, 6, and 7. (ZIP) [file ppat.1013058.s002.zip › S2 File/Original image-DOI 10.1371.journal.ppat.1009438/Figure7-Detailed raw data/7G/Fig7G-Merge-3.tif]

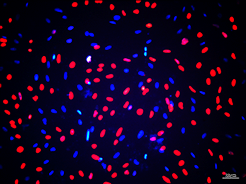

Supplement: S2 File — Underlying image data for Figures 1, 2, 4, 5, 6, and 7. (ZIP) [file ppat.1013058.s002.zip › S2 File/Original image-DOI 10.1371.journal.ppat.1009438/Figure7-Detailed raw data/7G/Fig7G-Merge-4.tif]

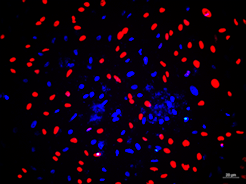

Supplement: S2 File — Underlying image data for Figures 1, 2, 4, 5, 6, and 7. (ZIP) [file ppat.1013058.s002.zip › S2 File/Original image-DOI 10.1371.journal.ppat.1009438/Figure7-Detailed raw data/7G/Fig7G-Merge-5.tif]

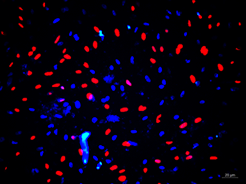

Supplement: S2 File — Underlying image data for Figures 1, 2, 4, 5, 6, and 7. (ZIP) [file ppat.1013058.s002.zip › S2 File/Original image-DOI 10.1371.journal.ppat.1009438/Figure7-Detailed raw data/7G/Fig7G-Merge-6.tif]

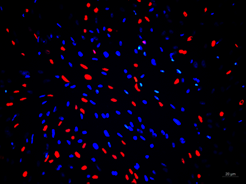

Supplement: S2 File — Underlying image data for Figures 1, 2, 4, 5, 6, and 7. (ZIP) [file ppat.1013058.s002.zip › S2 File/Original image-DOI 10.1371.journal.ppat.1009438/Figure7-Detailed raw data/7G/Fig7G-Merge-7.tif]

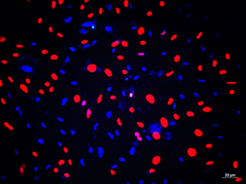

Supplement: S2 File — Underlying image data for Figures 1, 2, 4, 5, 6, and 7. (ZIP) [file ppat.1013058.s002.zip › S2 File/Original image-DOI 10.1371.journal.ppat.1009438/Figure7-Detailed raw data/7G/Fig7G-Merge-8.tif]

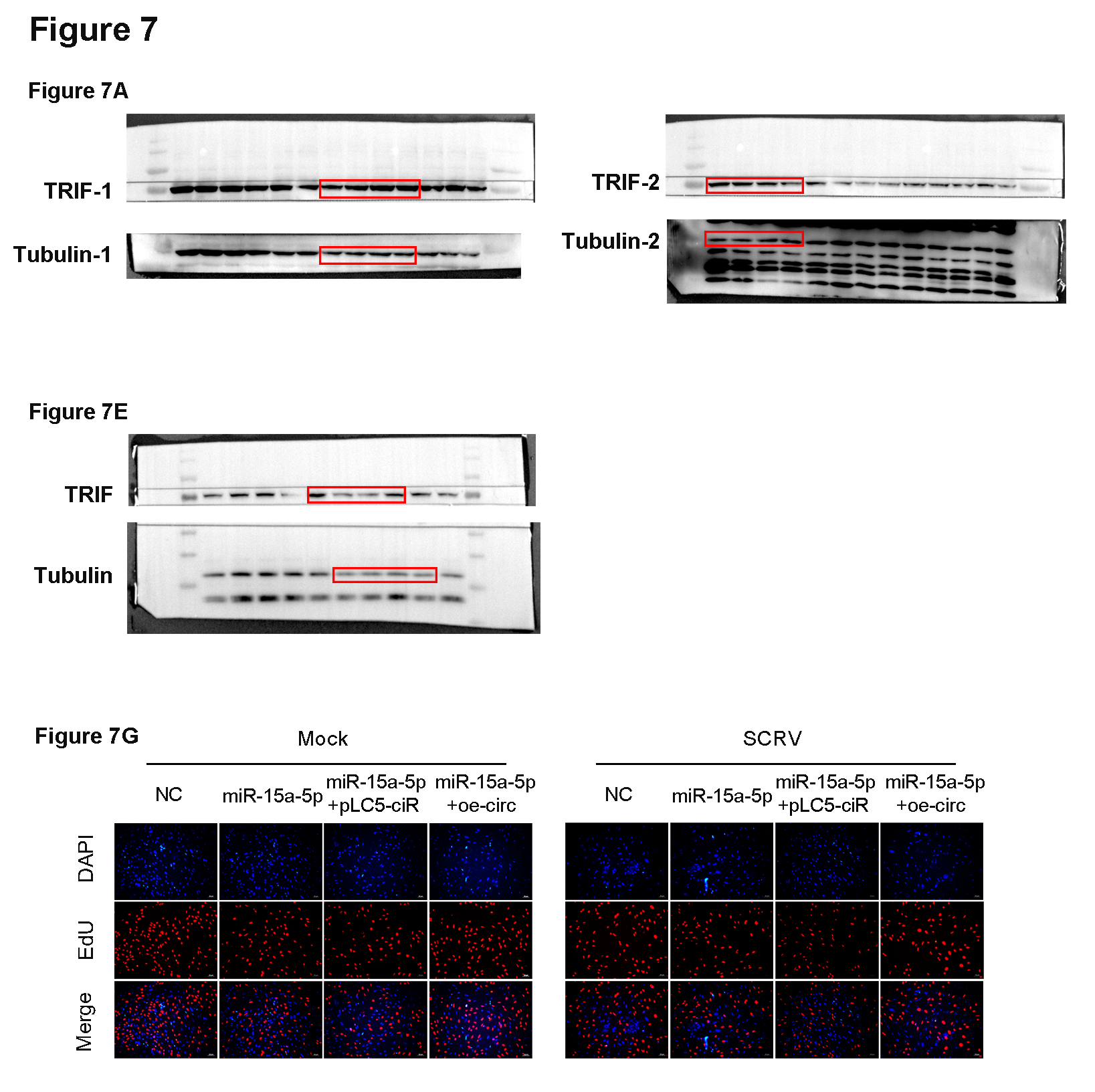

Supplement: S2 File — Underlying image data for Figures 1, 2, 4, 5, 6, and 7. (ZIP) [file ppat.1013058.s002.zip › S2 File/Original image-DOI 10.1371.journal.ppat.1009438/Figure7.tif]
