# Supplementary figures and images for: Correction: Circular RNA circDtx1 regulates IRF3-mediated antiviral immune responses through suppression of miR-15a-5p-dependent TRIF downregulation in teleost fish (part 1 of 2)
Source: PLoS Pathog. 2025 Apr 7;21(4):e1013058. doi: 10.1371/journal.ppat.1013058 (PMC11975111; doi:10.1371/journal.ppat.1013058)

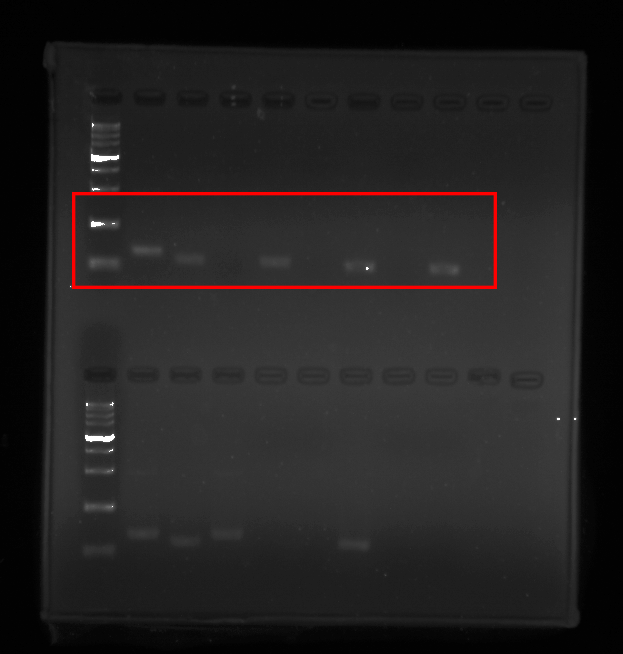

Supplement: S2 File — Underlying image data for Figures 1, 2, 4, 5, 6, and 7. (ZIP) [file ppat.1013058.s002.zip › S2 File/Original image-DOI 10.1371.journal.ppat.1009438/Figure1-Detailed raw data/1E/Fig1E-MIC.tif]

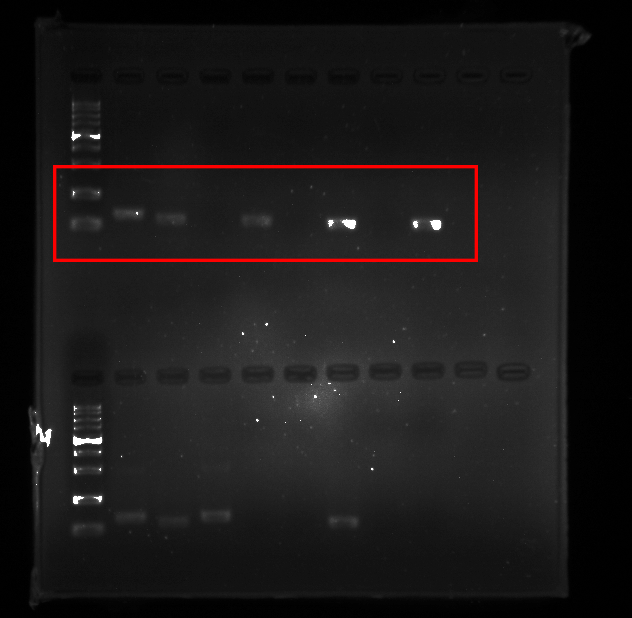

Supplement: S2 File — Underlying image data for Figures 1, 2, 4, 5, 6, and 7. (ZIP) [file ppat.1013058.s002.zip › S2 File/Original image-DOI 10.1371.journal.ppat.1009438/Figure1-Detailed raw data/1E/Fig1E-MKC.tif]

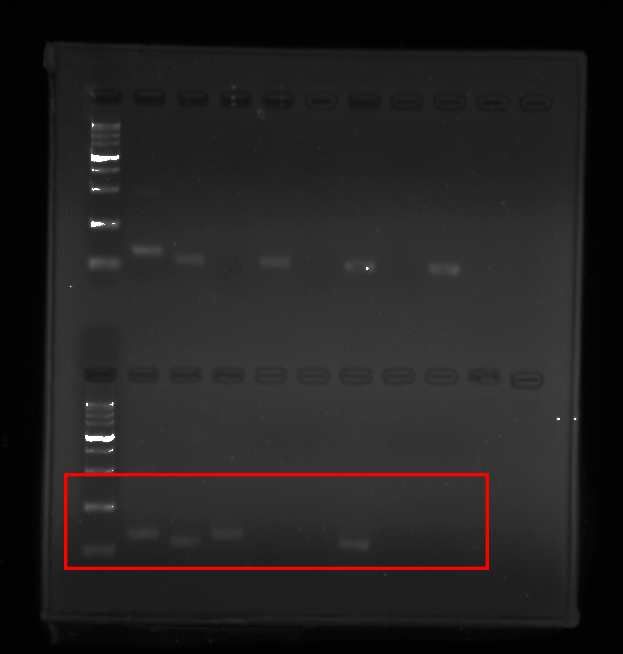

Supplement: S2 File — Underlying image data for Figures 1, 2, 4, 5, 6, and 7. (ZIP) [file ppat.1013058.s002.zip › S2 File/Original image-DOI 10.1371.journal.ppat.1009438/Figure1-Detailed raw data/1F/Fig1F-MIC.tif]

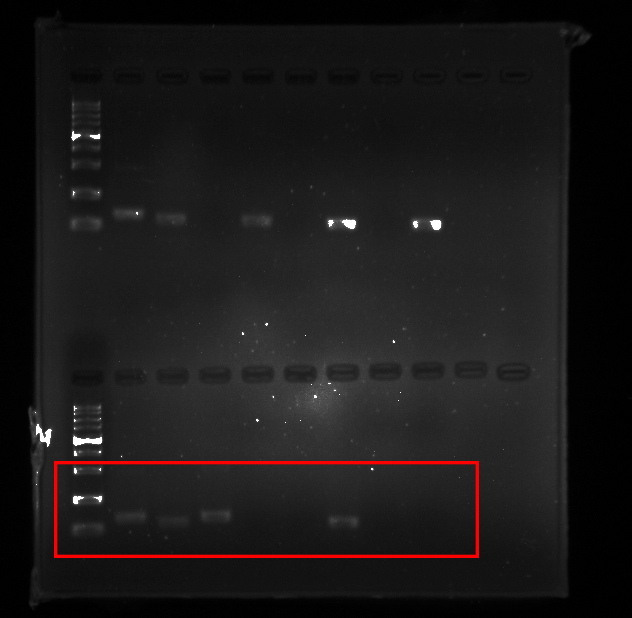

Supplement: S2 File — Underlying image data for Figures 1, 2, 4, 5, 6, and 7. (ZIP) [file ppat.1013058.s002.zip › S2 File/Original image-DOI 10.1371.journal.ppat.1009438/Figure1-Detailed raw data/1F/Fig1F-MKC.tif]

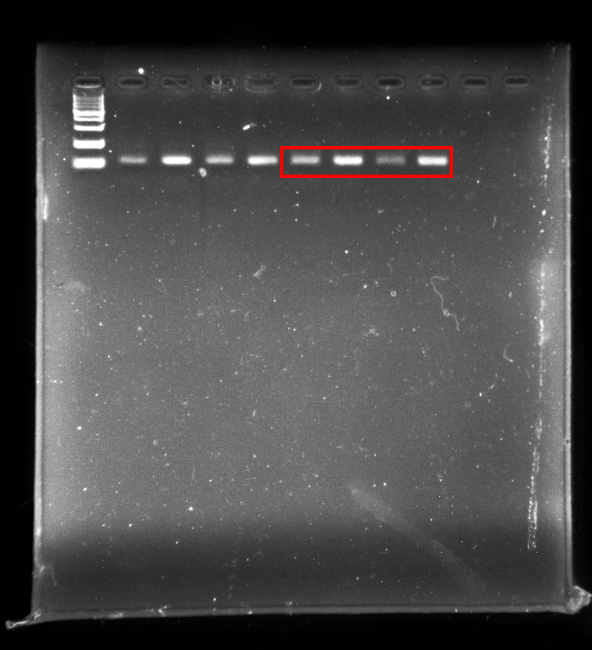

Supplement: S2 File — Underlying image data for Figures 1, 2, 4, 5, 6, and 7. (ZIP) [file ppat.1013058.s002.zip › S2 File/Original image-DOI 10.1371.journal.ppat.1009438/Figure1-Detailed raw data/1G/Fig1G-circDtx1.tif]

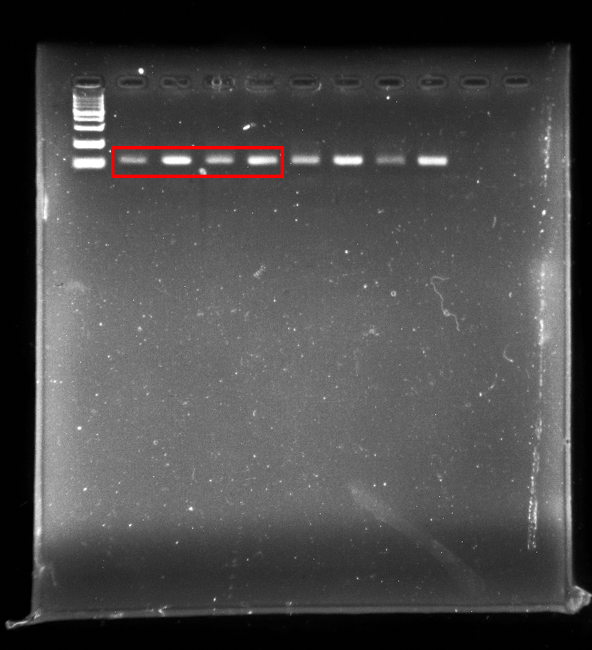

Supplement: S2 File — Underlying image data for Figures 1, 2, 4, 5, 6, and 7. (ZIP) [file ppat.1013058.s002.zip › S2 File/Original image-DOI 10.1371.journal.ppat.1009438/Figure1-Detailed raw data/1G/Fig1G-GAPDH.tif]

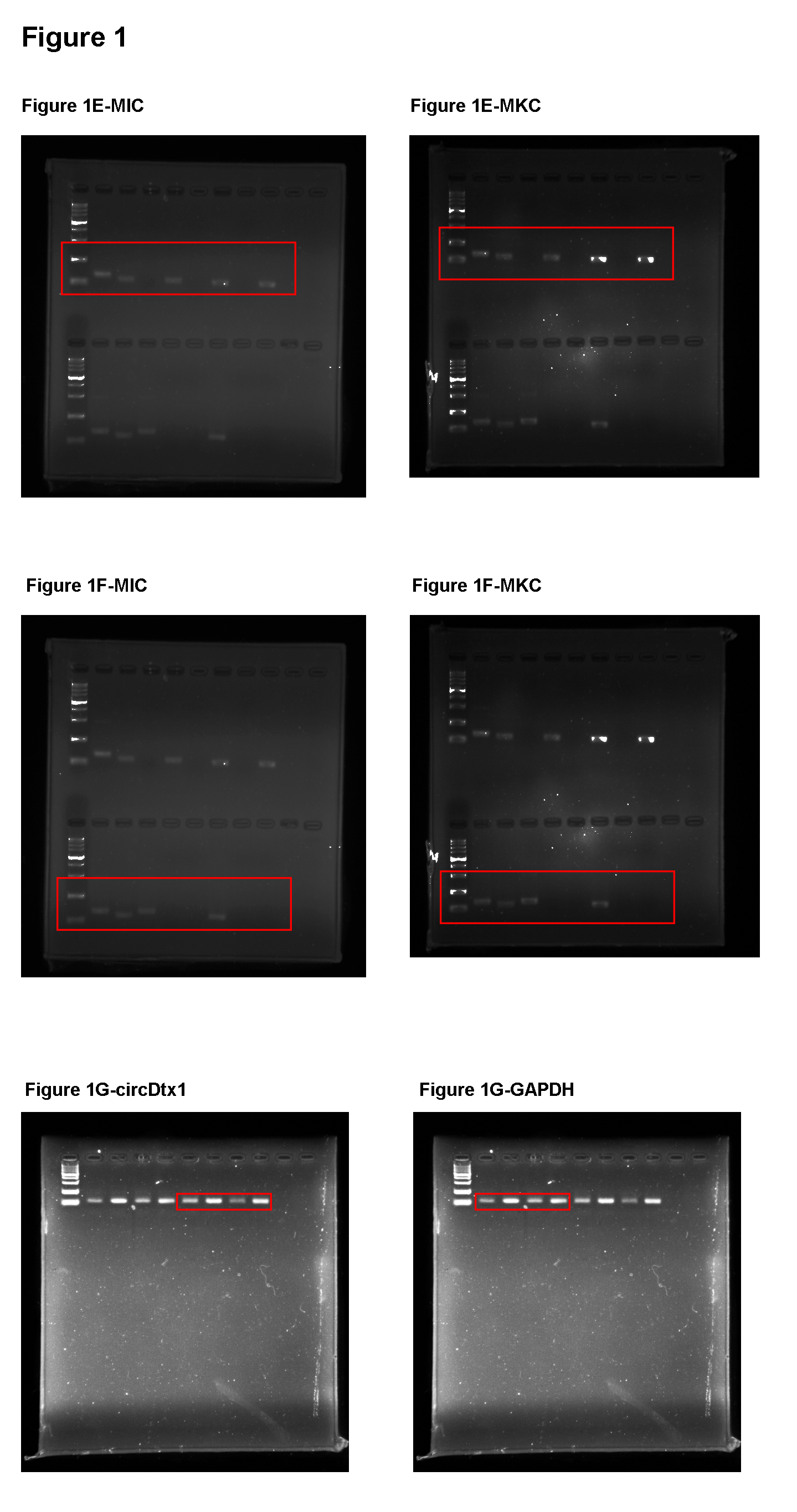

Supplement: S2 File — Underlying image data for Figures 1, 2, 4, 5, 6, and 7. (ZIP) [file ppat.1013058.s002.zip › S2 File/Original image-DOI 10.1371.journal.ppat.1009438/Figure1.tif]

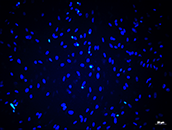

Supplement: S2 File — Underlying image data for Figures 1, 2, 4, 5, 6, and 7. (ZIP) [file ppat.1013058.s002.zip › S2 File/Original image-DOI 10.1371.journal.ppat.1009438/Figure2-Detailed raw data/2F/Fig2F-DAPI-1.tif]

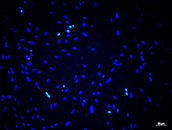

Supplement: S2 File — Underlying image data for Figures 1, 2, 4, 5, 6, and 7. (ZIP) [file ppat.1013058.s002.zip › S2 File/Original image-DOI 10.1371.journal.ppat.1009438/Figure2-Detailed raw data/2F/Fig2F-DAPI-2.tif]

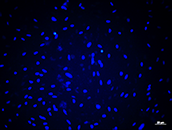

Supplement: S2 File — Underlying image data for Figures 1, 2, 4, 5, 6, and 7. (ZIP) [file ppat.1013058.s002.zip › S2 File/Original image-DOI 10.1371.journal.ppat.1009438/Figure2-Detailed raw data/2F/Fig2F-DAPI-3.tif]

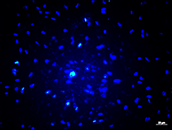

Supplement: S2 File — Underlying image data for Figures 1, 2, 4, 5, 6, and 7. (ZIP) [file ppat.1013058.s002.zip › S2 File/Original image-DOI 10.1371.journal.ppat.1009438/Figure2-Detailed raw data/2F/Fig2F-DAPI-4.tif]

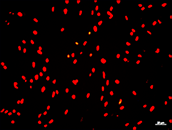

Supplement: S2 File — Underlying image data for Figures 1, 2, 4, 5, 6, and 7. (ZIP) [file ppat.1013058.s002.zip › S2 File/Original image-DOI 10.1371.journal.ppat.1009438/Figure2-Detailed raw data/2F/Fig2F-EdU-1.tif]

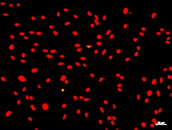

Supplement: S2 File — Underlying image data for Figures 1, 2, 4, 5, 6, and 7. (ZIP) [file ppat.1013058.s002.zip › S2 File/Original image-DOI 10.1371.journal.ppat.1009438/Figure2-Detailed raw data/2F/Fig2F-EdU-2.tif]

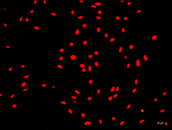

Supplement: S2 File — Underlying image data for Figures 1, 2, 4, 5, 6, and 7. (ZIP) [file ppat.1013058.s002.zip › S2 File/Original image-DOI 10.1371.journal.ppat.1009438/Figure2-Detailed raw data/2F/Fig2F-EdU-3.tif]

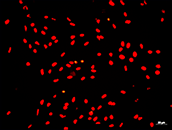

Supplement: S2 File — Underlying image data for Figures 1, 2, 4, 5, 6, and 7. (ZIP) [file ppat.1013058.s002.zip › S2 File/Original image-DOI 10.1371.journal.ppat.1009438/Figure2-Detailed raw data/2F/Fig2F-EdU-4.tif]

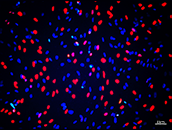

Supplement: S2 File — Underlying image data for Figures 1, 2, 4, 5, 6, and 7. (ZIP) [file ppat.1013058.s002.zip › S2 File/Original image-DOI 10.1371.journal.ppat.1009438/Figure2-Detailed raw data/2F/Fig2F-Merge-1.tif]

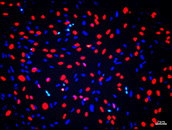

Supplement: S2 File — Underlying image data for Figures 1, 2, 4, 5, 6, and 7. (ZIP) [file ppat.1013058.s002.zip › S2 File/Original image-DOI 10.1371.journal.ppat.1009438/Figure2-Detailed raw data/2F/Fig2F-Merge-2.tif]

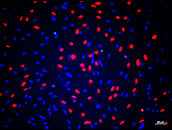

Supplement: S2 File — Underlying image data for Figures 1, 2, 4, 5, 6, and 7. (ZIP) [file ppat.1013058.s002.zip › S2 File/Original image-DOI 10.1371.journal.ppat.1009438/Figure2-Detailed raw data/2F/Fig2F-Merge-3.tif]

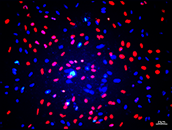

Supplement: S2 File — Underlying image data for Figures 1, 2, 4, 5, 6, and 7. (ZIP) [file ppat.1013058.s002.zip › S2 File/Original image-DOI 10.1371.journal.ppat.1009438/Figure2-Detailed raw data/2F/Fig2F-Merge-4.tif]

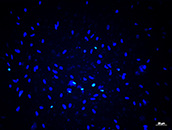

Supplement: S2 File — Underlying image data for Figures 1, 2, 4, 5, 6, and 7. (ZIP) [file ppat.1013058.s002.zip › S2 File/Original image-DOI 10.1371.journal.ppat.1009438/Figure2-Detailed raw data/2G/Fig2G-DAPI-1.tif]

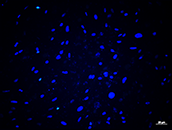

Supplement: S2 File — Underlying image data for Figures 1, 2, 4, 5, 6, and 7. (ZIP) [file ppat.1013058.s002.zip › S2 File/Original image-DOI 10.1371.journal.ppat.1009438/Figure2-Detailed raw data/2G/Fig2G-DAPI-2.tif]

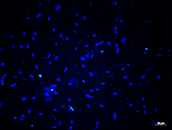

Supplement: S2 File — Underlying image data for Figures 1, 2, 4, 5, 6, and 7. (ZIP) [file ppat.1013058.s002.zip › S2 File/Original image-DOI 10.1371.journal.ppat.1009438/Figure2-Detailed raw data/2G/Fig2G-DAPI-3.tif]

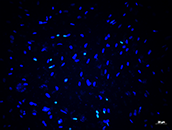

Supplement: S2 File — Underlying image data for Figures 1, 2, 4, 5, 6, and 7. (ZIP) [file ppat.1013058.s002.zip › S2 File/Original image-DOI 10.1371.journal.ppat.1009438/Figure2-Detailed raw data/2G/Fig2G-DAPI-4.tif]

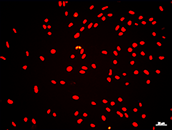

Supplement: S2 File — Underlying image data for Figures 1, 2, 4, 5, 6, and 7. (ZIP) [file ppat.1013058.s002.zip › S2 File/Original image-DOI 10.1371.journal.ppat.1009438/Figure2-Detailed raw data/2G/Fig2G-EdU-1.tif]

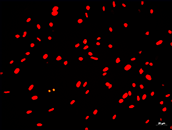

Supplement: S2 File — Underlying image data for Figures 1, 2, 4, 5, 6, and 7. (ZIP) [file ppat.1013058.s002.zip › S2 File/Original image-DOI 10.1371.journal.ppat.1009438/Figure2-Detailed raw data/2G/Fig2G-EdU-2.tif]

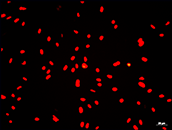

Supplement: S2 File — Underlying image data for Figures 1, 2, 4, 5, 6, and 7. (ZIP) [file ppat.1013058.s002.zip › S2 File/Original image-DOI 10.1371.journal.ppat.1009438/Figure2-Detailed raw data/2G/Fig2G-EdU-3.tif]

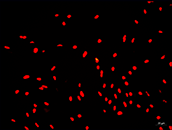

Supplement: S2 File — Underlying image data for Figures 1, 2, 4, 5, 6, and 7. (ZIP) [file ppat.1013058.s002.zip › S2 File/Original image-DOI 10.1371.journal.ppat.1009438/Figure2-Detailed raw data/2G/Fig2G-EdU-4.tif]

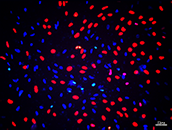

Supplement: S2 File — Underlying image data for Figures 1, 2, 4, 5, 6, and 7. (ZIP) [file ppat.1013058.s002.zip › S2 File/Original image-DOI 10.1371.journal.ppat.1009438/Figure2-Detailed raw data/2G/Fig2G-Merge-1.tif]

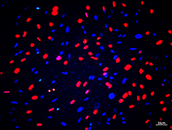

Supplement: S2 File — Underlying image data for Figures 1, 2, 4, 5, 6, and 7. (ZIP) [file ppat.1013058.s002.zip › S2 File/Original image-DOI 10.1371.journal.ppat.1009438/Figure2-Detailed raw data/2G/Fig2G-Merge-2.tif]

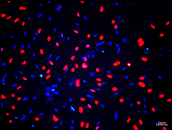

Supplement: S2 File — Underlying image data for Figures 1, 2, 4, 5, 6, and 7. (ZIP) [file ppat.1013058.s002.zip › S2 File/Original image-DOI 10.1371.journal.ppat.1009438/Figure2-Detailed raw data/2G/Fig2G-Merge-3.tif]

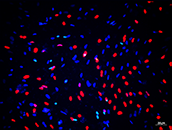

Supplement: S2 File — Underlying image data for Figures 1, 2, 4, 5, 6, and 7. (ZIP) [file ppat.1013058.s002.zip › S2 File/Original image-DOI 10.1371.journal.ppat.1009438/Figure2-Detailed raw data/2G/Fig2G-Merge-4.tif]

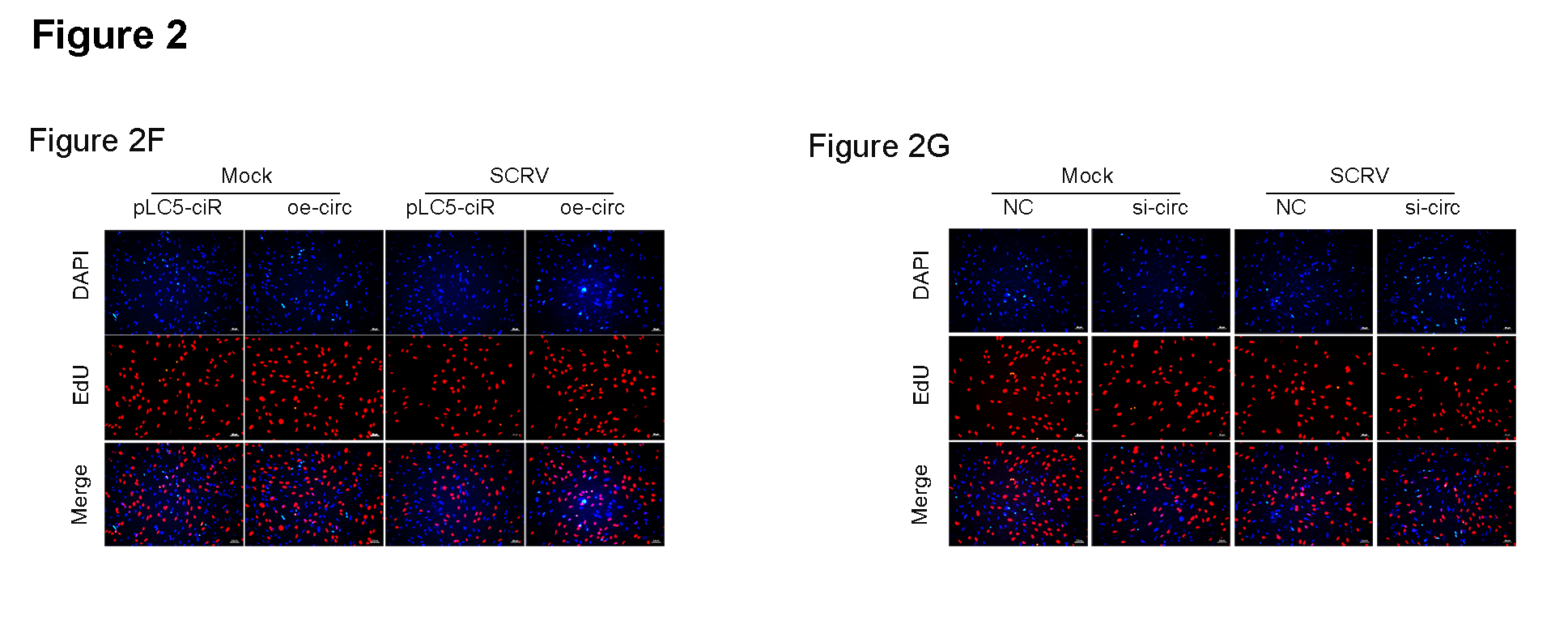

Supplement: S2 File — Underlying image data for Figures 1, 2, 4, 5, 6, and 7. (ZIP) [file ppat.1013058.s002.zip › S2 File/Original image-DOI 10.1371.journal.ppat.1009438/Figure2.tif]

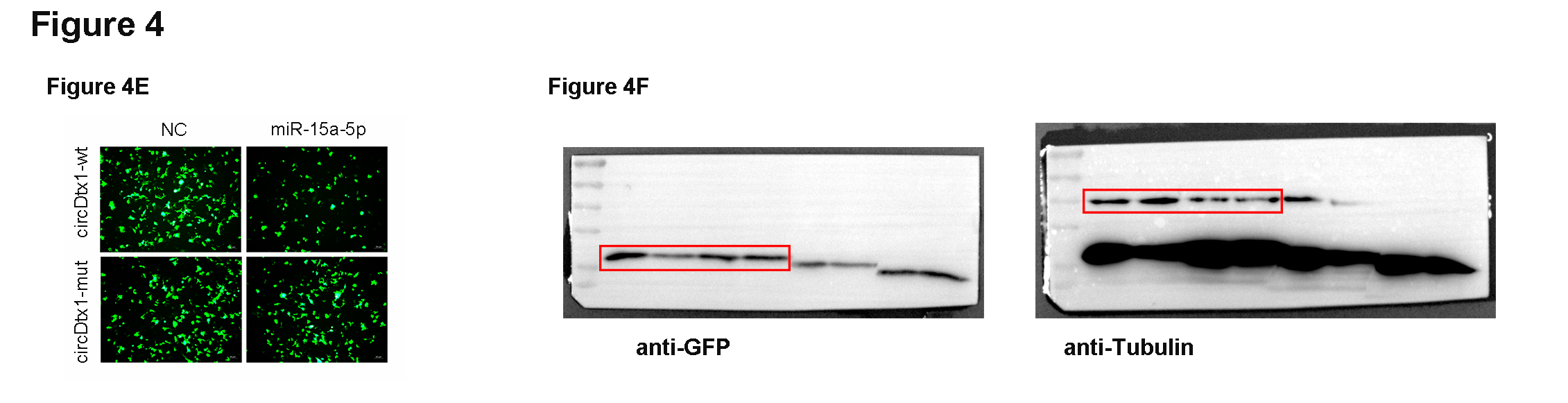

Supplement: S2 File — Underlying image data for Figures 1, 2, 4, 5, 6, and 7. (ZIP) [file ppat.1013058.s002.zip › S2 File/Original image-DOI 10.1371.journal.ppat.1009438/Figure3.tif]

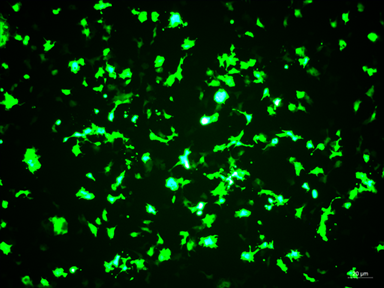

Supplement: S2 File — Underlying image data for Figures 1, 2, 4, 5, 6, and 7. (ZIP) [file ppat.1013058.s002.zip › S2 File/Original image-DOI 10.1371.journal.ppat.1009438/Figure4-Detailed raw data/4E/Fig4E-1.tif]

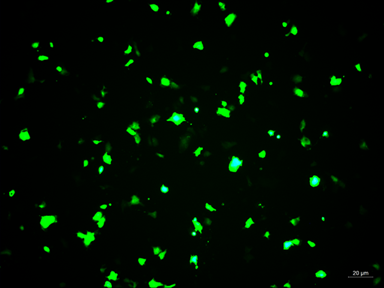

Supplement: S2 File — Underlying image data for Figures 1, 2, 4, 5, 6, and 7. (ZIP) [file ppat.1013058.s002.zip › S2 File/Original image-DOI 10.1371.journal.ppat.1009438/Figure4-Detailed raw data/4E/Fig4E-2.tif]

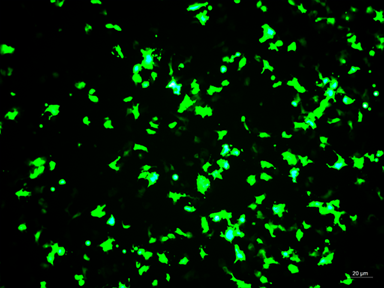

Supplement: S2 File — Underlying image data for Figures 1, 2, 4, 5, 6, and 7. (ZIP) [file ppat.1013058.s002.zip › S2 File/Original image-DOI 10.1371.journal.ppat.1009438/Figure4-Detailed raw data/4E/Fig4E-3.tif]

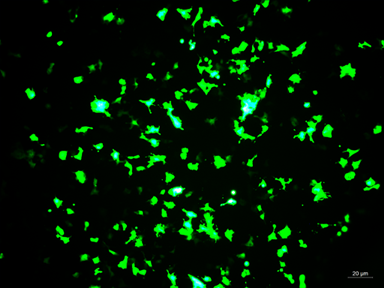

Supplement: S2 File — Underlying image data for Figures 1, 2, 4, 5, 6, and 7. (ZIP) [file ppat.1013058.s002.zip › S2 File/Original image-DOI 10.1371.journal.ppat.1009438/Figure4-Detailed raw data/4E/Fig4E-4.tif]

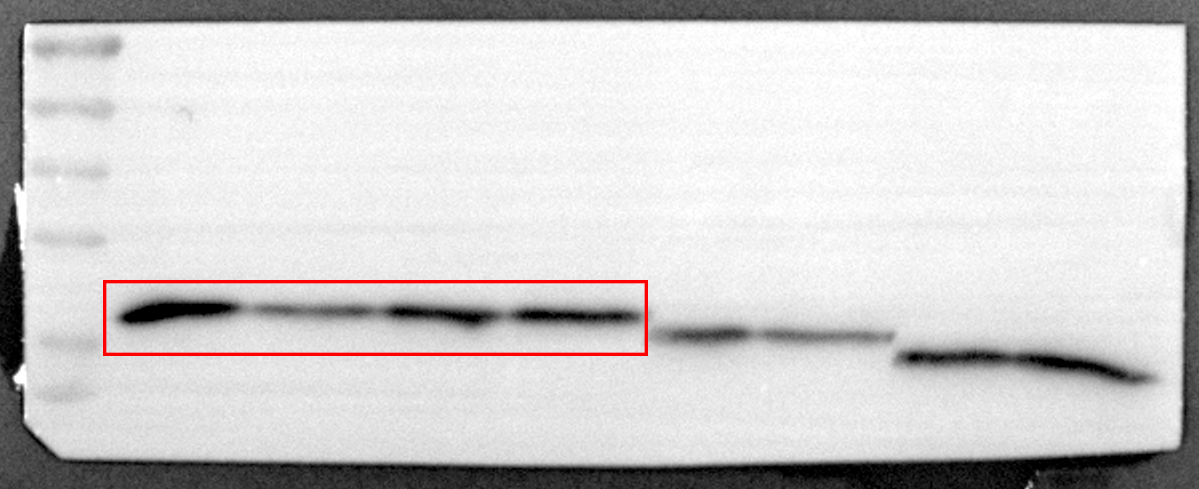

Supplement: S2 File — Underlying image data for Figures 1, 2, 4, 5, 6, and 7. (ZIP) [file ppat.1013058.s002.zip › S2 File/Original image-DOI 10.1371.journal.ppat.1009438/Figure4-Detailed raw data/4F/Fig4F-Anti-GFP.tif]

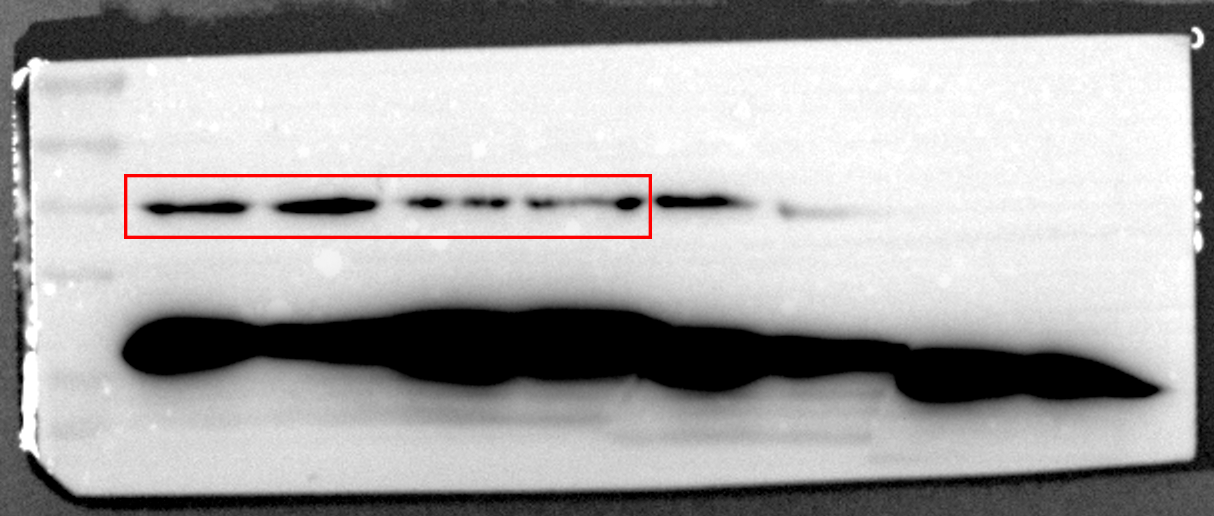

Supplement: S2 File — Underlying image data for Figures 1, 2, 4, 5, 6, and 7. (ZIP) [file ppat.1013058.s002.zip › S2 File/Original image-DOI 10.1371.journal.ppat.1009438/Figure4-Detailed raw data/4F/Fig4F-Tubulin.tif]

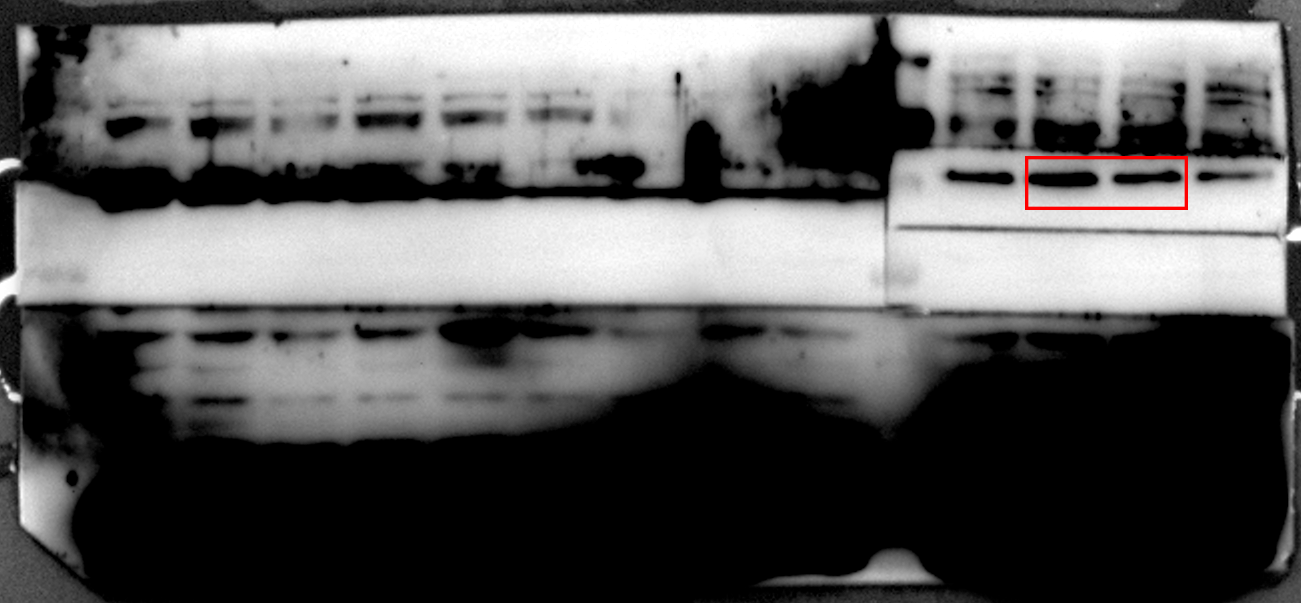

Supplement: S2 File — Underlying image data for Figures 1, 2, 4, 5, 6, and 7. (ZIP) [file ppat.1013058.s002.zip › S2 File/Original image-DOI 10.1371.journal.ppat.1009438/Figure5-Detailed raw data/5C/Fig5C-TRIF.tif]

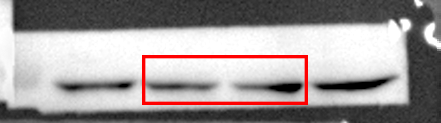

Supplement: S2 File — Underlying image data for Figures 1, 2, 4, 5, 6, and 7. (ZIP) [file ppat.1013058.s002.zip › S2 File/Original image-DOI 10.1371.journal.ppat.1009438/Figure5-Detailed raw data/5C/Fig5C-Tubulin.tif]

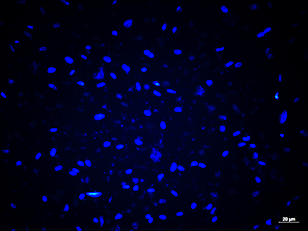

Supplement: S2 File — Underlying image data for Figures 1, 2, 4, 5, 6, and 7. (ZIP) [file ppat.1013058.s002.zip › S2 File/Original image-DOI 10.1371.journal.ppat.1009438/Figure5-Detailed raw data/5G/Fig5G-DAPI-1.tif]

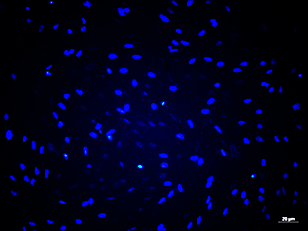

Supplement: S2 File — Underlying image data for Figures 1, 2, 4, 5, 6, and 7. (ZIP) [file ppat.1013058.s002.zip › S2 File/Original image-DOI 10.1371.journal.ppat.1009438/Figure5-Detailed raw data/5G/Fig5G-DAPI-2.tif]

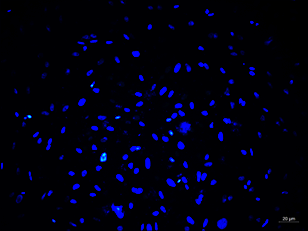

Supplement: S2 File — Underlying image data for Figures 1, 2, 4, 5, 6, and 7. (ZIP) [file ppat.1013058.s002.zip › S2 File/Original image-DOI 10.1371.journal.ppat.1009438/Figure5-Detailed raw data/5G/Fig5G-DAPI-3.tif]

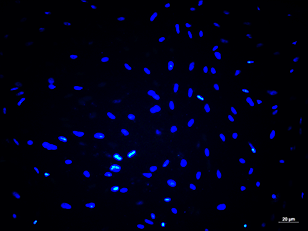

Supplement: S2 File — Underlying image data for Figures 1, 2, 4, 5, 6, and 7. (ZIP) [file ppat.1013058.s002.zip › S2 File/Original image-DOI 10.1371.journal.ppat.1009438/Figure5-Detailed raw data/5G/Fig5G-DAPI-4.tif]

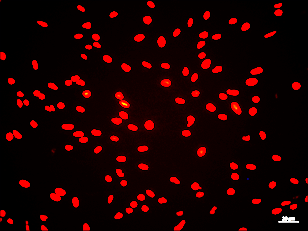

Supplement: S2 File — Underlying image data for Figures 1, 2, 4, 5, 6, and 7. (ZIP) [file ppat.1013058.s002.zip › S2 File/Original image-DOI 10.1371.journal.ppat.1009438/Figure5-Detailed raw data/5G/Fig5G-EdU-1.tif]

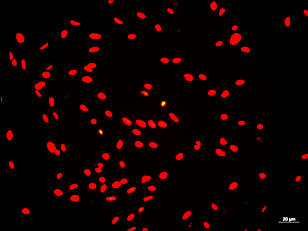

Supplement: S2 File — Underlying image data for Figures 1, 2, 4, 5, 6, and 7. (ZIP) [file ppat.1013058.s002.zip › S2 File/Original image-DOI 10.1371.journal.ppat.1009438/Figure5-Detailed raw data/5G/Fig5G-EdU-2.tif]

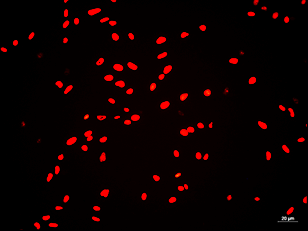

Supplement: S2 File — Underlying image data for Figures 1, 2, 4, 5, 6, and 7. (ZIP) [file ppat.1013058.s002.zip › S2 File/Original image-DOI 10.1371.journal.ppat.1009438/Figure5-Detailed raw data/5G/Fig5G-EdU-3.tif]

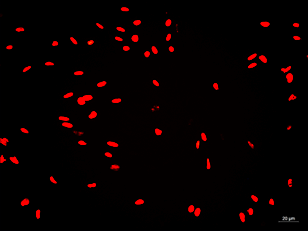

Supplement: S2 File — Underlying image data for Figures 1, 2, 4, 5, 6, and 7. (ZIP) [file ppat.1013058.s002.zip › S2 File/Original image-DOI 10.1371.journal.ppat.1009438/Figure5-Detailed raw data/5G/Fig5G-EdU-4.tif]

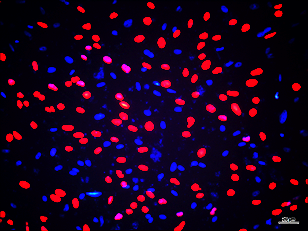

Supplement: S2 File — Underlying image data for Figures 1, 2, 4, 5, 6, and 7. (ZIP) [file ppat.1013058.s002.zip › S2 File/Original image-DOI 10.1371.journal.ppat.1009438/Figure5-Detailed raw data/5G/Fig5G-Merge-1.tif]

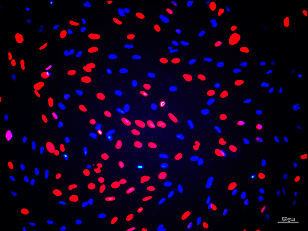

Supplement: S2 File — Underlying image data for Figures 1, 2, 4, 5, 6, and 7. (ZIP) [file ppat.1013058.s002.zip › S2 File/Original image-DOI 10.1371.journal.ppat.1009438/Figure5-Detailed raw data/5G/Fig5G-Merge-2.tif]

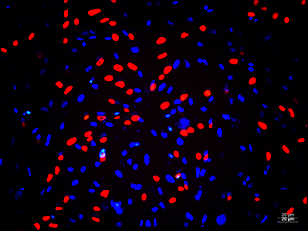

Supplement: S2 File — Underlying image data for Figures 1, 2, 4, 5, 6, and 7. (ZIP) [file ppat.1013058.s002.zip › S2 File/Original image-DOI 10.1371.journal.ppat.1009438/Figure5-Detailed raw data/5G/Fig5G-Merge-3.tif]

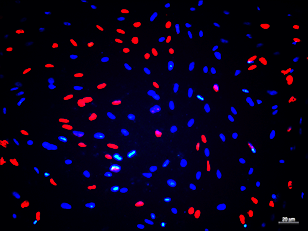

Supplement: S2 File — Underlying image data for Figures 1, 2, 4, 5, 6, and 7. (ZIP) [file ppat.1013058.s002.zip › S2 File/Original image-DOI 10.1371.journal.ppat.1009438/Figure5-Detailed raw data/5G/Fig5G-Merge-4.tif]

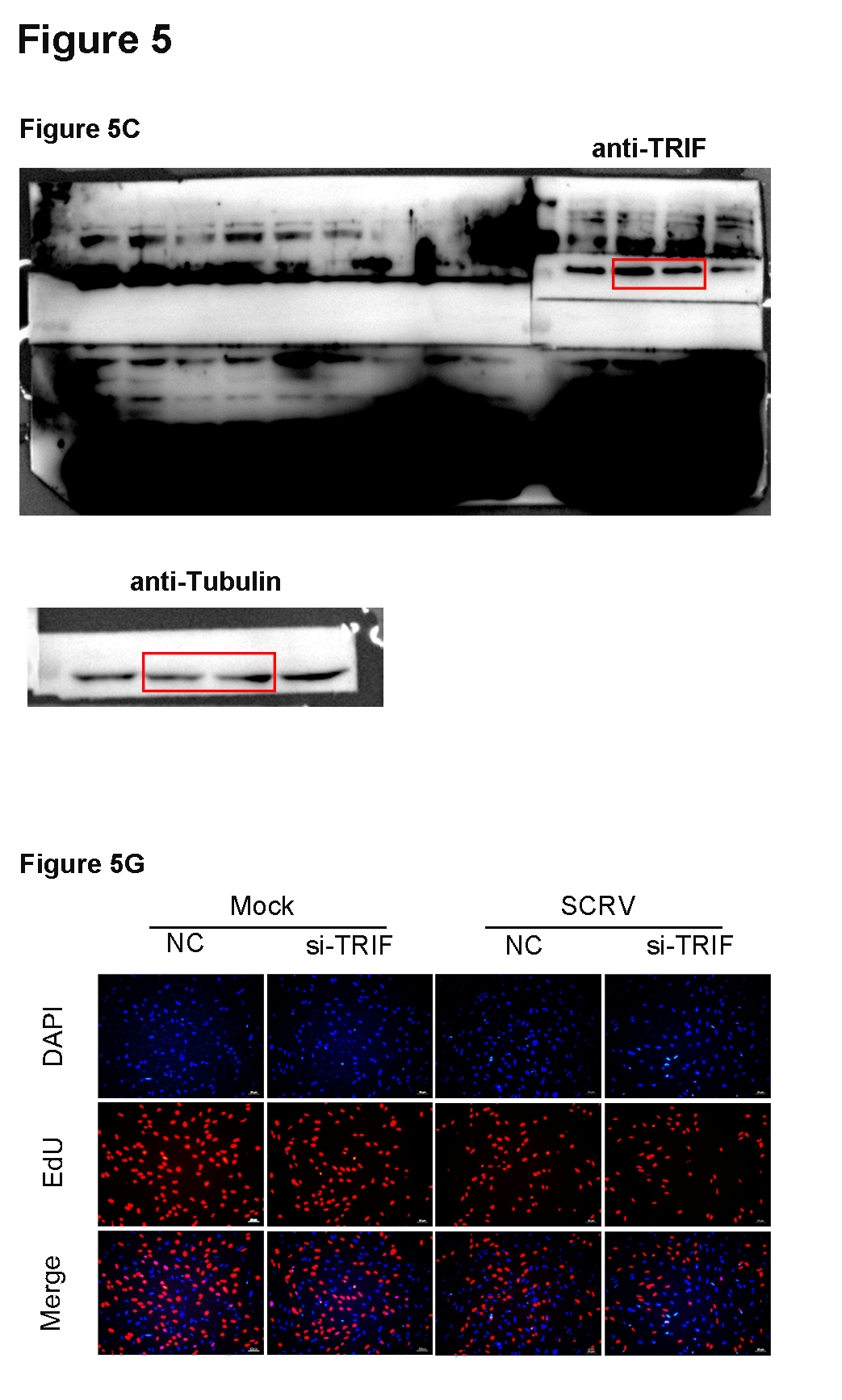

Supplement: S2 File — Underlying image data for Figures 1, 2, 4, 5, 6, and 7. (ZIP) [file ppat.1013058.s002.zip › S2 File/Original image-DOI 10.1371.journal.ppat.1009438/Figure5.tif]

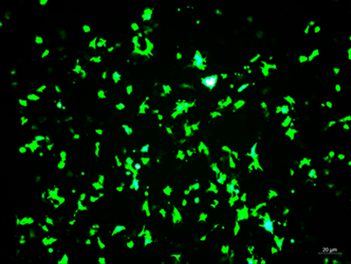

Supplement: S2 File — Underlying image data for Figures 1, 2, 4, 5, 6, and 7. (ZIP) [file ppat.1013058.s002.zip › S2 File/Original image-DOI 10.1371.journal.ppat.1009438/Figure6-Detailed raw data/6C/Fig6C-1.tif]

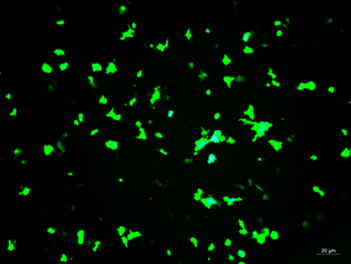

Supplement: S2 File — Underlying image data for Figures 1, 2, 4, 5, 6, and 7. (ZIP) [file ppat.1013058.s002.zip › S2 File/Original image-DOI 10.1371.journal.ppat.1009438/Figure6-Detailed raw data/6C/Fig6C-2.tif]

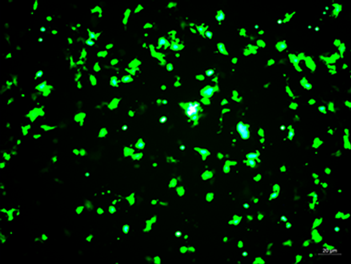

Supplement: S2 File — Underlying image data for Figures 1, 2, 4, 5, 6, and 7. (ZIP) [file ppat.1013058.s002.zip › S2 File/Original image-DOI 10.1371.journal.ppat.1009438/Figure6-Detailed raw data/6C/Fig6C-3.tif]

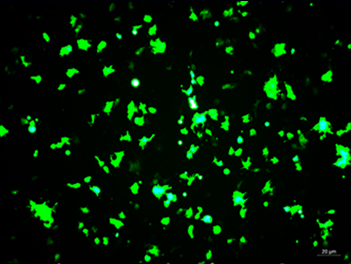

Supplement: S2 File — Underlying image data for Figures 1, 2, 4, 5, 6, and 7. (ZIP) [file ppat.1013058.s002.zip › S2 File/Original image-DOI 10.1371.journal.ppat.1009438/Figure6-Detailed raw data/6C/Fig6C-4.tif]

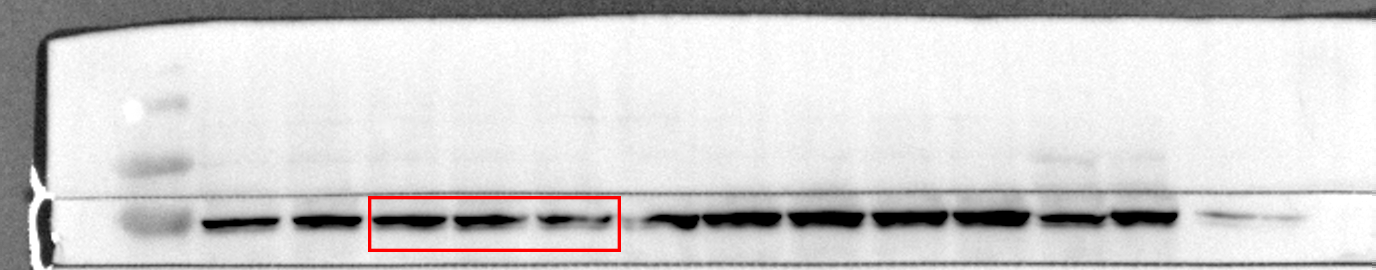

Supplement: S2 File — Underlying image data for Figures 1, 2, 4, 5, 6, and 7. (ZIP) [file ppat.1013058.s002.zip › S2 File/Original image-DOI 10.1371.journal.ppat.1009438/Figure6-Detailed raw data/6D/Fig6D-TRIF-1.tif]

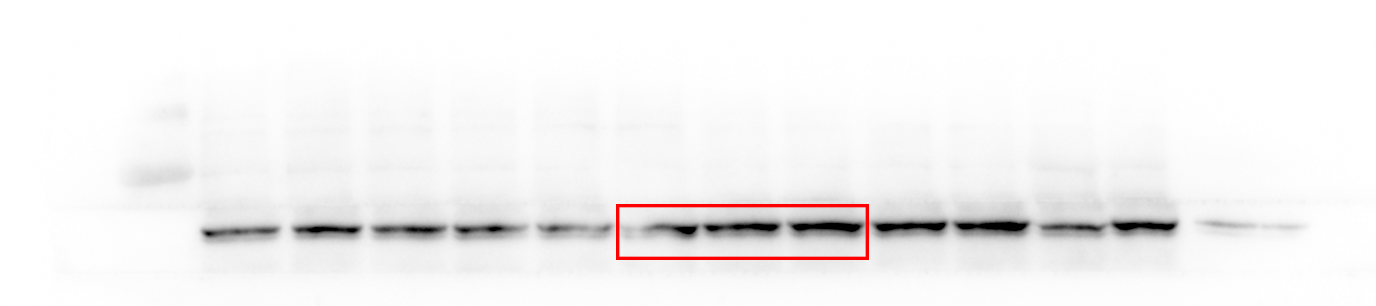

Supplement: S2 File — Underlying image data for Figures 1, 2, 4, 5, 6, and 7. (ZIP) [file ppat.1013058.s002.zip › S2 File/Original image-DOI 10.1371.journal.ppat.1009438/Figure6-Detailed raw data/6D/Fig6D-TRIF-2.tif]

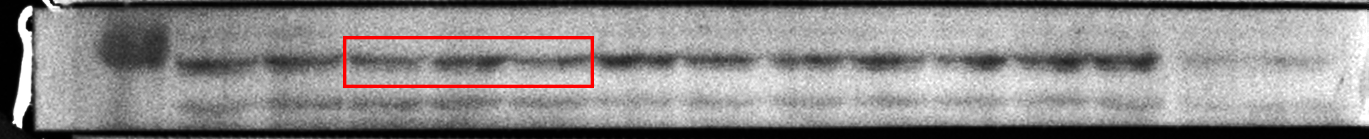

Supplement: S2 File — Underlying image data for Figures 1, 2, 4, 5, 6, and 7. (ZIP) [file ppat.1013058.s002.zip › S2 File/Original image-DOI 10.1371.journal.ppat.1009438/Figure6-Detailed raw data/6D/Fig6D-Tubulin-1.tif]

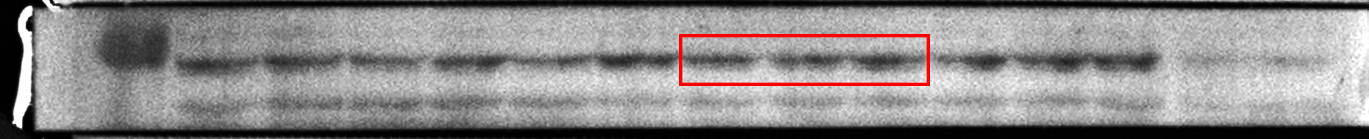

Supplement: S2 File — Underlying image data for Figures 1, 2, 4, 5, 6, and 7. (ZIP) [file ppat.1013058.s002.zip › S2 File/Original image-DOI 10.1371.journal.ppat.1009438/Figure6-Detailed raw data/6D/Fig6D-Tubulin-2.tif]

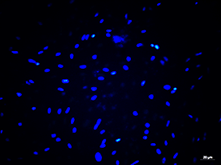

Supplement: S2 File — Underlying image data for Figures 1, 2, 4, 5, 6, and 7. (ZIP) [file ppat.1013058.s002.zip › S2 File/Original image-DOI 10.1371.journal.ppat.1009438/Figure6-Detailed raw data/6I/Fig6I-DAPI-1.tif]

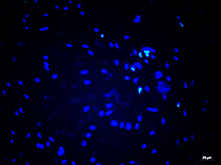

Supplement: S2 File — Underlying image data for Figures 1, 2, 4, 5, 6, and 7. (ZIP) [file ppat.1013058.s002.zip › S2 File/Original image-DOI 10.1371.journal.ppat.1009438/Figure6-Detailed raw data/6I/Fig6I-DAPI-2.tif]

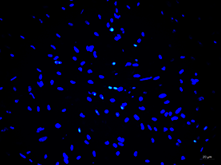

Supplement: S2 File — Underlying image data for Figures 1, 2, 4, 5, 6, and 7. (ZIP) [file ppat.1013058.s002.zip › S2 File/Original image-DOI 10.1371.journal.ppat.1009438/Figure6-Detailed raw data/6I/Fig6I-DAPI-3.tif]

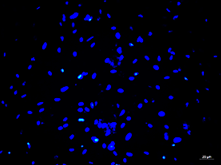

Supplement: S2 File — Underlying image data for Figures 1, 2, 4, 5, 6, and 7. (ZIP) [file ppat.1013058.s002.zip › S2 File/Original image-DOI 10.1371.journal.ppat.1009438/Figure6-Detailed raw data/6I/Fig6I-DAPI-4.tif]

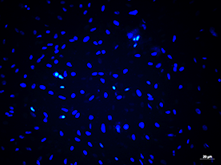

Supplement: S2 File — Underlying image data for Figures 1, 2, 4, 5, 6, and 7. (ZIP) [file ppat.1013058.s002.zip › S2 File/Original image-DOI 10.1371.journal.ppat.1009438/Figure6-Detailed raw data/6I/Fig6I-DAPI-5.tif]

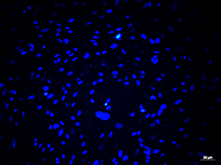

Supplement: S2 File — Underlying image data for Figures 1, 2, 4, 5, 6, and 7. (ZIP) [file ppat.1013058.s002.zip › S2 File/Original image-DOI 10.1371.journal.ppat.1009438/Figure6-Detailed raw data/6I/Fig6I-DAPI-6.tif]

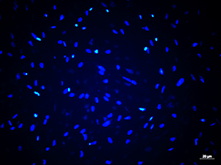

Supplement: S2 File — Underlying image data for Figures 1, 2, 4, 5, 6, and 7. (ZIP) [file ppat.1013058.s002.zip › S2 File/Original image-DOI 10.1371.journal.ppat.1009438/Figure6-Detailed raw data/6I/Fig6I-DAPI-7.tif]

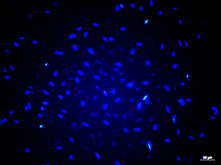

Supplement: S2 File — Underlying image data for Figures 1, 2, 4, 5, 6, and 7. (ZIP) [file ppat.1013058.s002.zip › S2 File/Original image-DOI 10.1371.journal.ppat.1009438/Figure6-Detailed raw data/6I/Fig6I-DAPI-8.tif]

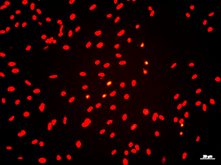

Supplement: S2 File — Underlying image data for Figures 1, 2, 4, 5, 6, and 7. (ZIP) [file ppat.1013058.s002.zip › S2 File/Original image-DOI 10.1371.journal.ppat.1009438/Figure6-Detailed raw data/6I/Fig6I-EdU-1.tif]

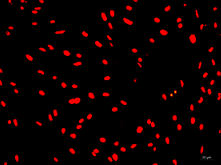

Supplement: S2 File — Underlying image data for Figures 1, 2, 4, 5, 6, and 7. (ZIP) [file ppat.1013058.s002.zip › S2 File/Original image-DOI 10.1371.journal.ppat.1009438/Figure6-Detailed raw data/6I/Fig6I-EdU-2.tif]

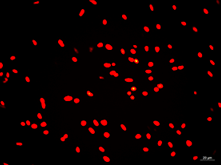

Supplement: S2 File — Underlying image data for Figures 1, 2, 4, 5, 6, and 7. (ZIP) [file ppat.1013058.s002.zip › S2 File/Original image-DOI 10.1371.journal.ppat.1009438/Figure6-Detailed raw data/6I/Fig6I-EdU-3.tif]

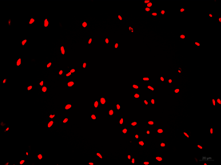

Supplement: S2 File — Underlying image data for Figures 1, 2, 4, 5, 6, and 7. (ZIP) [file ppat.1013058.s002.zip › S2 File/Original image-DOI 10.1371.journal.ppat.1009438/Figure6-Detailed raw data/6I/Fig6I-EdU-4.tif]

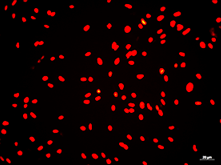

Supplement: S2 File — Underlying image data for Figures 1, 2, 4, 5, 6, and 7. (ZIP) [file ppat.1013058.s002.zip › S2 File/Original image-DOI 10.1371.journal.ppat.1009438/Figure6-Detailed raw data/6I/Fig6I-EdU-5.tif]

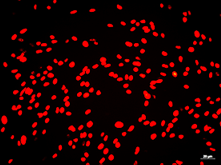

Supplement: S2 File — Underlying image data for Figures 1, 2, 4, 5, 6, and 7. (ZIP) [file ppat.1013058.s002.zip › S2 File/Original image-DOI 10.1371.journal.ppat.1009438/Figure6-Detailed raw data/6I/Fig6I-EdU-6.tif]

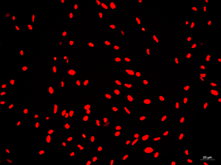

Supplement: S2 File — Underlying image data for Figures 1, 2, 4, 5, 6, and 7. (ZIP) [file ppat.1013058.s002.zip › S2 File/Original image-DOI 10.1371.journal.ppat.1009438/Figure6-Detailed raw data/6I/Fig6I-EdU-7.tif]

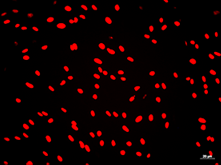

Supplement: S2 File — Underlying image data for Figures 1, 2, 4, 5, 6, and 7. (ZIP) [file ppat.1013058.s002.zip › S2 File/Original image-DOI 10.1371.journal.ppat.1009438/Figure6-Detailed raw data/6I/Fig6I-EdU-8.tif]

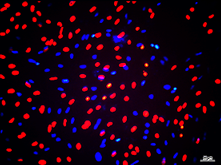

Supplement: S2 File — Underlying image data for Figures 1, 2, 4, 5, 6, and 7. (ZIP) [file ppat.1013058.s002.zip › S2 File/Original image-DOI 10.1371.journal.ppat.1009438/Figure6-Detailed raw data/6I/Fig6I-Merge-1.tif]

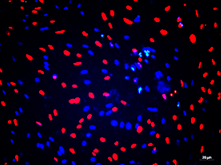

Supplement: S2 File — Underlying image data for Figures 1, 2, 4, 5, 6, and 7. (ZIP) [file ppat.1013058.s002.zip › S2 File/Original image-DOI 10.1371.journal.ppat.1009438/Figure6-Detailed raw data/6I/Fig6I-Merge-2.tif]

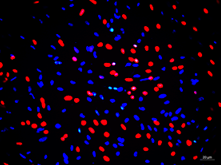

Supplement: S2 File — Underlying image data for Figures 1, 2, 4, 5, 6, and 7. (ZIP) [file ppat.1013058.s002.zip › S2 File/Original image-DOI 10.1371.journal.ppat.1009438/Figure6-Detailed raw data/6I/Fig6I-Merge-3.tif]

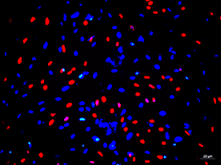

Supplement: S2 File — Underlying image data for Figures 1, 2, 4, 5, 6, and 7. (ZIP) [file ppat.1013058.s002.zip › S2 File/Original image-DOI 10.1371.journal.ppat.1009438/Figure6-Detailed raw data/6I/Fig6I-Merge-4.tif]

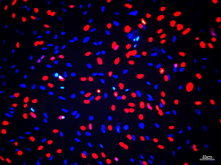

Supplement: S2 File — Underlying image data for Figures 1, 2, 4, 5, 6, and 7. (ZIP) [file ppat.1013058.s002.zip › S2 File/Original image-DOI 10.1371.journal.ppat.1009438/Figure6-Detailed raw data/6I/Fig6I-Merge-5.tif]

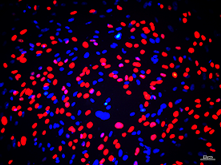

Supplement: S2 File — Underlying image data for Figures 1, 2, 4, 5, 6, and 7. (ZIP) [file ppat.1013058.s002.zip › S2 File/Original image-DOI 10.1371.journal.ppat.1009438/Figure6-Detailed raw data/6I/Fig6I-Merge-6.tif]

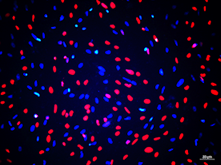

Supplement: S2 File — Underlying image data for Figures 1, 2, 4, 5, 6, and 7. (ZIP) [file ppat.1013058.s002.zip › S2 File/Original image-DOI 10.1371.journal.ppat.1009438/Figure6-Detailed raw data/6I/Fig6I-Merge-7.tif]

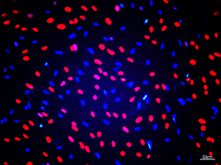

Supplement: S2 File — Underlying image data for Figures 1, 2, 4, 5, 6, and 7. (ZIP) [file ppat.1013058.s002.zip › S2 File/Original image-DOI 10.1371.journal.ppat.1009438/Figure6-Detailed raw data/6I/Fig6I-Merge-8.tif]

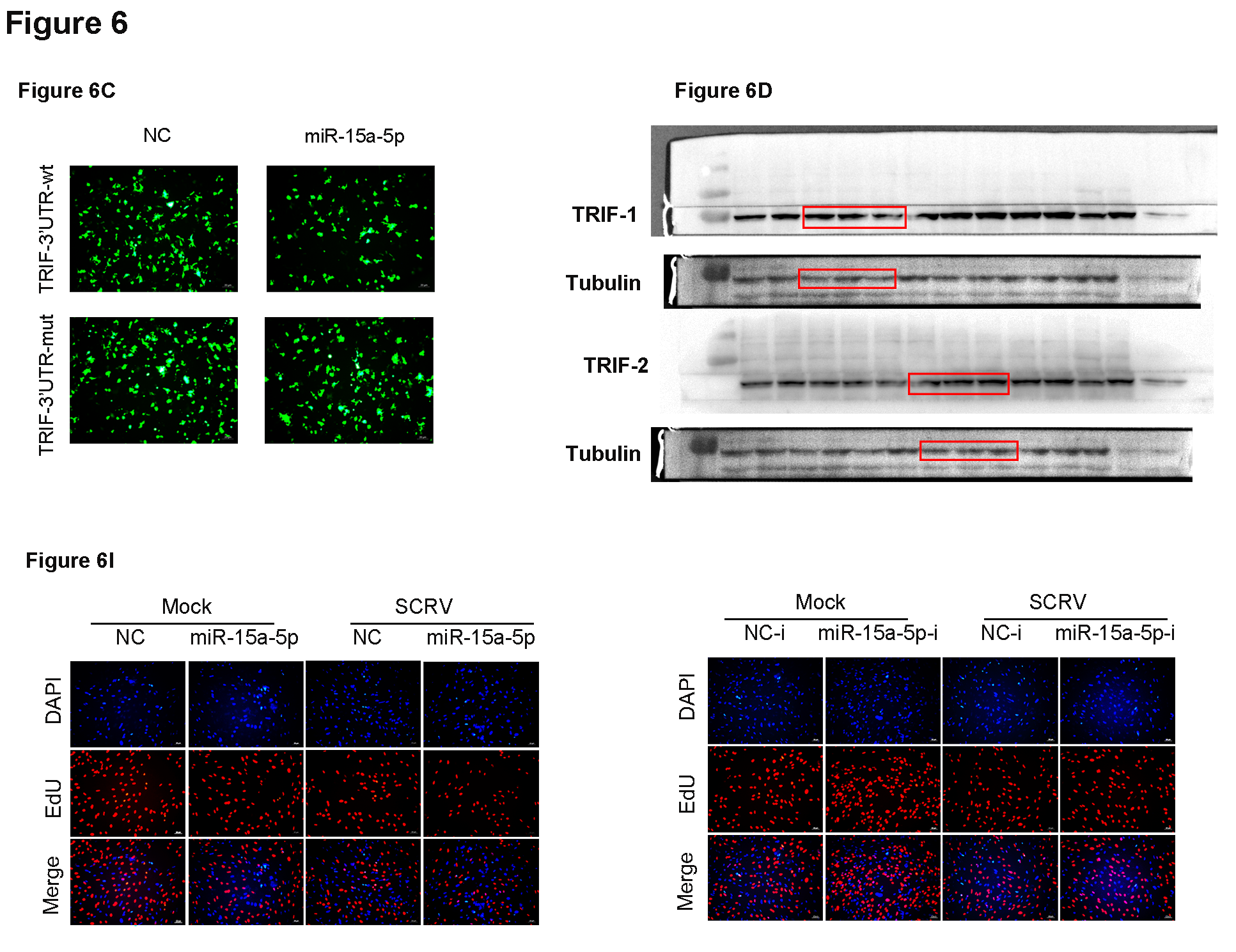

Supplement: S2 File — Underlying image data for Figures 1, 2, 4, 5, 6, and 7. (ZIP) [file ppat.1013058.s002.zip › S2 File/Original image-DOI 10.1371.journal.ppat.1009438/Figure6.tif]

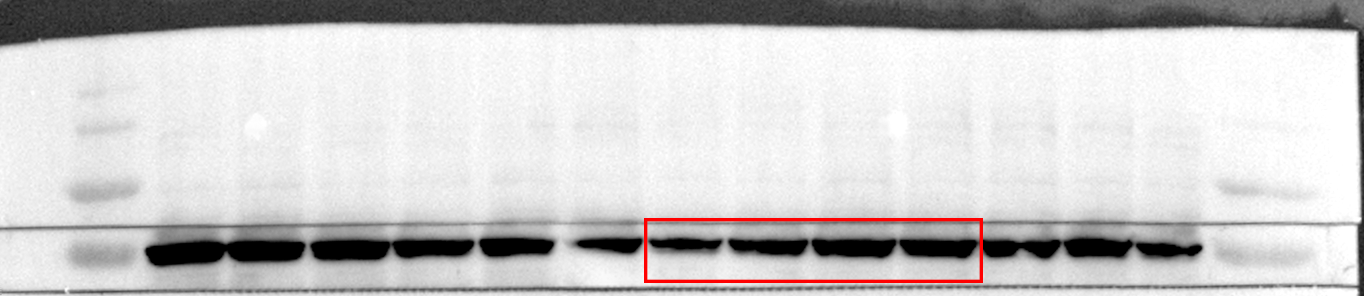

Supplement: S2 File — Underlying image data for Figures 1, 2, 4, 5, 6, and 7. (ZIP) [file ppat.1013058.s002.zip › S2 File/Original image-DOI 10.1371.journal.ppat.1009438/Figure7-Detailed raw data/7A/Fig7A-TRIF-1.tif]

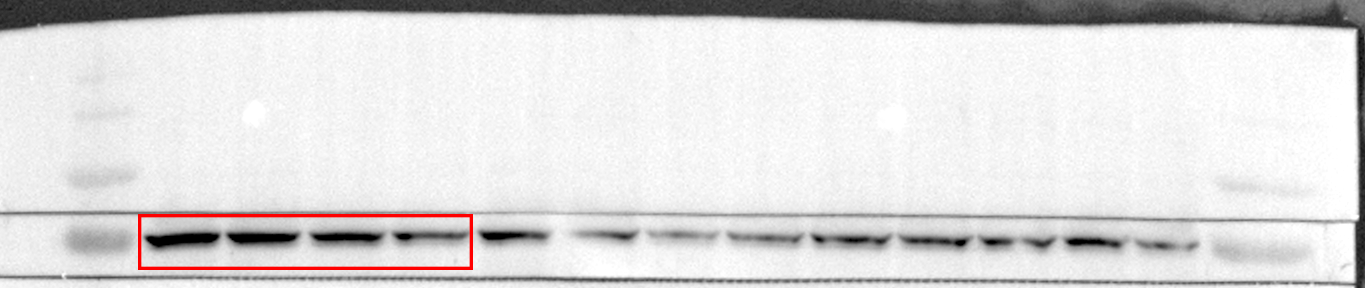

Supplement: S2 File — Underlying image data for Figures 1, 2, 4, 5, 6, and 7. (ZIP) [file ppat.1013058.s002.zip › S2 File/Original image-DOI 10.1371.journal.ppat.1009438/Figure7-Detailed raw data/7A/Fig7A-TRIF-2.tif]

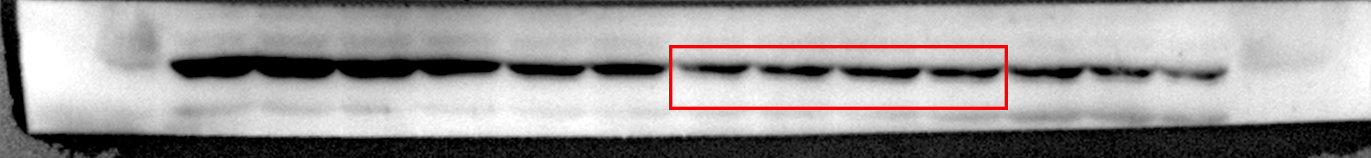

Supplement: S2 File — Underlying image data for Figures 1, 2, 4, 5, 6, and 7. (ZIP) [file ppat.1013058.s002.zip › S2 File/Original image-DOI 10.1371.journal.ppat.1009438/Figure7-Detailed raw data/7A/Fig7A-Tubulin-1.tif]

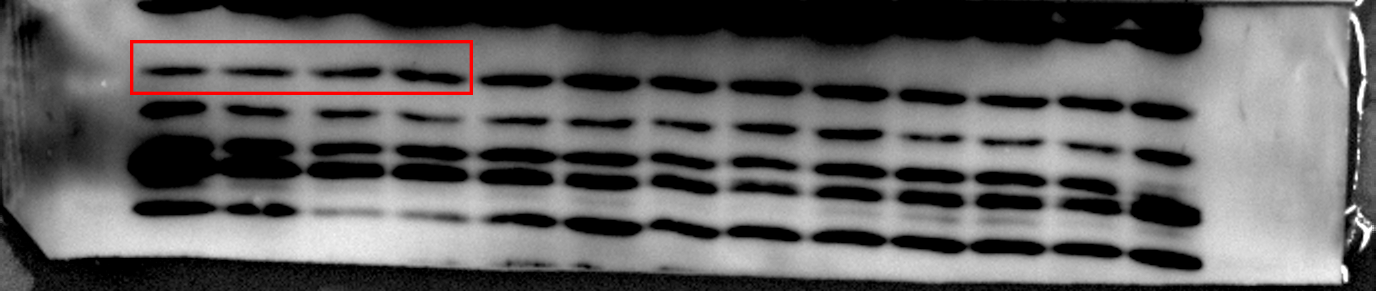

Supplement: S2 File — Underlying image data for Figures 1, 2, 4, 5, 6, and 7. (ZIP) [file ppat.1013058.s002.zip › S2 File/Original image-DOI 10.1371.journal.ppat.1009438/Figure7-Detailed raw data/7A/Fig7A-Tubulin-2.tif]

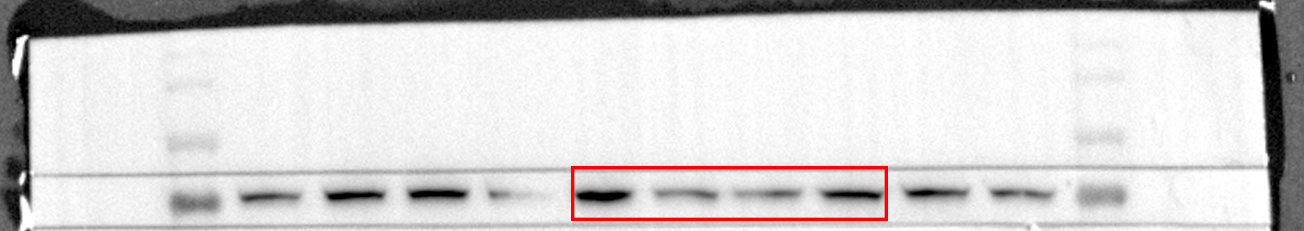

Supplement: S2 File — Underlying image data for Figures 1, 2, 4, 5, 6, and 7. (ZIP) [file ppat.1013058.s002.zip › S2 File/Original image-DOI 10.1371.journal.ppat.1009438/Figure7-Detailed raw data/7E/Fig7E-TRIF.tif]

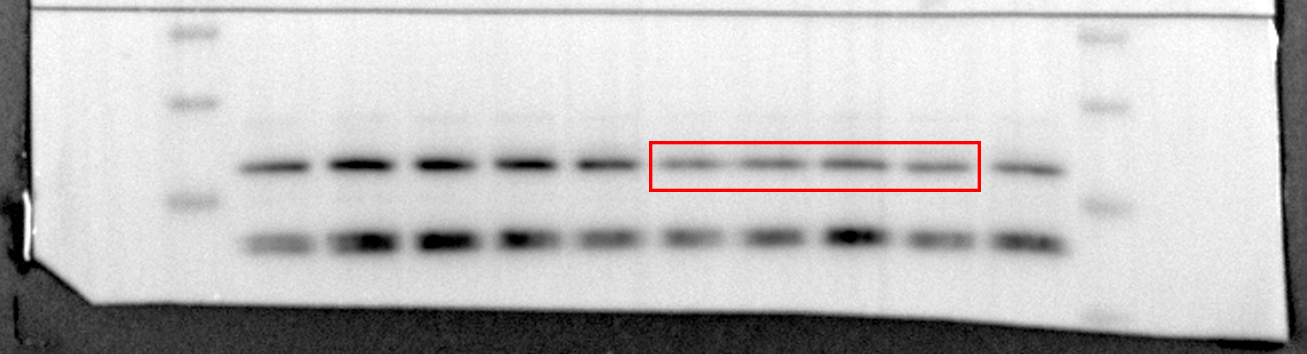

Supplement: S2 File — Underlying image data for Figures 1, 2, 4, 5, 6, and 7. (ZIP) [file ppat.1013058.s002.zip › S2 File/Original image-DOI 10.1371.journal.ppat.1009438/Figure7-Detailed raw data/7E/Fig7E-Tubulin.tif]

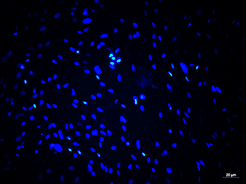

Supplement: S2 File — Underlying image data for Figures 1, 2, 4, 5, 6, and 7. (ZIP) [file ppat.1013058.s002.zip › S2 File/Original image-DOI 10.1371.journal.ppat.1009438/Figure7-Detailed raw data/7G/Fig7G-DAPI-1.tif]

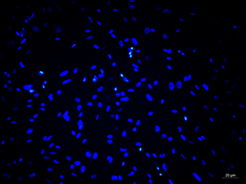

Supplement: S2 File — Underlying image data for Figures 1, 2, 4, 5, 6, and 7. (ZIP) [file ppat.1013058.s002.zip › S2 File/Original image-DOI 10.1371.journal.ppat.1009438/Figure7-Detailed raw data/7G/Fig7G-DAPI-2.tif]

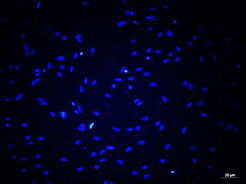

Supplement: S2 File — Underlying image data for Figures 1, 2, 4, 5, 6, and 7. (ZIP) [file ppat.1013058.s002.zip › S2 File/Original image-DOI 10.1371.journal.ppat.1009438/Figure7-Detailed raw data/7G/Fig7G-DAPI-3.tif]

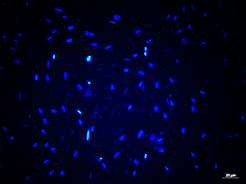

Supplement: S2 File — Underlying image data for Figures 1, 2, 4, 5, 6, and 7. (ZIP) [file ppat.1013058.s002.zip › S2 File/Original image-DOI 10.1371.journal.ppat.1009438/Figure7-Detailed raw data/7G/Fig7G-DAPI-4.tif]

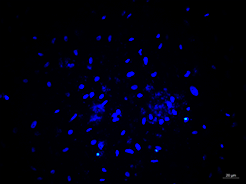

Supplement: S2 File — Underlying image data for Figures 1, 2, 4, 5, 6, and 7. (ZIP) [file ppat.1013058.s002.zip › S2 File/Original image-DOI 10.1371.journal.ppat.1009438/Figure7-Detailed raw data/7G/Fig7G-DAPI-5.tif]

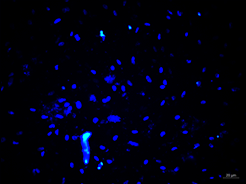

Supplement: S2 File — Underlying image data for Figures 1, 2, 4, 5, 6, and 7. (ZIP) [file ppat.1013058.s002.zip › S2 File/Original image-DOI 10.1371.journal.ppat.1009438/Figure7-Detailed raw data/7G/Fig7G-DAPI-6.tif]

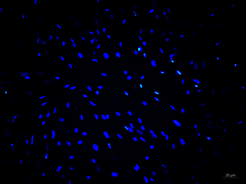

Supplement: S2 File — Underlying image data for Figures 1, 2, 4, 5, 6, and 7. (ZIP) [file ppat.1013058.s002.zip › S2 File/Original image-DOI 10.1371.journal.ppat.1009438/Figure7-Detailed raw data/7G/Fig7G-DAPI-7.tif]
